# Voucher-verified checklist of Colombian Pacific marine fishes with emphasis on material deposited in Colombian museums

**DOI:** 10.3897/zookeys.1272.141461

**Published:** 2026-03-12

**Authors:** Jose Tavera, Beatriz S. Beltrán-León, Andrea Polanco F, Luis A. Zapata, Fernando A. Zapata, Arturo Acero P

**Affiliations:** 1 Departamento de Biología, Universidad del Valle, Cali, Colombia Universidad del Valle Cali Colombia; 2 Fundación Biodiversa Colombia, Calle 117 # 70H - 16, Bogotá DC, Colombia Fundación Biodiversa Colombia Bogotá Colombia; 3 WWF, Cali, Colombia Universidad Nacional de Colombia sede Caribe Santa Marta Colombia; 4 Instituto de Estudios en Ciencias del Mar, Cecimar, Universidad Nacional de Colombia sede Caribe, Santa Marta, Colombia WWF Cali Colombia

**Keywords:** Biodiversity, distribution, Eastern Tropical Pacific, ichthyology

## Abstract

A checklist of marine fishes from the Colombian Pacific is presented based on an appraisal of local inventories published in peer-reviewed journals and validation of records with voucher specimens deposited in Colombian marine fish collections and photographic records. Unpublished information collected over several years by the authors is also included. An exhaustive re-evaluation of the taxonomy of all species present in the area was also carried out. As a result, 727 species were confirmed, representing close to 50% of the 1,468 shallow water fish species reported from the Eastern Tropical Pacific (ETP). The diversity of marine fishes in Colombia was classified into three classes, 60 orders, 149 families, and 412 genera. Of these classes, only one species belongs to Myxini (hagfishes), 64 to Chondrichthyes (sharks, rays, and chimaeras), and 663 to Actinopterygii (bony fishes). In addition, 35 new records are presented for the Pacific of Colombia. Finally, all this diversity is characterized according to different ecological variables such as depth and bottom preference, among others, with the following results: 43% of all the species are benthopelagic, 54% of which are predominantly soft bottom dwellers. A total of 283 bony fishes (39% of all the species) are considered to have commercial value or are involved in some trade. Additionally, a list of 77 species that have only been recorded in larval stages is also presented. This comprehensive list provides a valuable resource for future research, conservation efforts, and fisheries management in the Colombian Pacific.

## Introduction

Although there is a long and notable history of ichthyological work in the Eastern Tropical Pacific (ETP) region dating back to the 19^th^ century (Jenyns 1842; Jordan 1895; Jordan and Evermann 1896–1900), the first comprehensive studies included very little material from Colombia. Those studies focused on collections from Panama to Mexico, including the Galapagos Islands (Jordan and Evermann 1896–1900; Gilbert and Starks 1904; Meek and Hildebrand 1923–1928), and Peru (Hildebrand 1946). Subsequent work continued to be restricted to specific countries or regions such as Peru (Chirichigno 1978; Chirichigno and Vélez 1998; Chirichigno and Cornejo 2001), Ecuador (Béarez 1996), the Galapagos Islands (Grove and Lavenberg 1997; Victor et al. 2024), Mexico as a whole (Castro-Aguirre et al. 1999; Fricke et al. 2024a) or just the Gulf of California (Thomson et al. 1979, 2000; De la Cruz-Agüero et al. 1997), and recently a list of the fishes of the northeastern Pacific from Canada to the tip of Baja California (Love et al. 2021), while Colombian species were mostly only recorded within major regional works (Allen and Robertson 1994; Fischer et al. 1995a, 1995b).

The first records of fish fauna in Colombian Pacific waters were documented by foreign scientists, mainly through material collected by U.S. and British expeditions. A chronological summary of the most notable ones includes the Eastern Pacific Expeditions of the U.S. National Museum of Natural History on the steamer ‘Albatross’ (Jordan and Bollman 1890; Kendall and Radcliffe 1912), the visit of the British yacht ‘St. George’ to Gorgona Island in 1927 (Garth 1948), the Allan Hancock Foundation Expeditions (1931–1938) in Pacific waters aboard the ‘Velero III’ (Myers and Reid 1936; Ginsburg 1938; Herald 1940; Myers and Wade 1941, 1942, 1946; Wade 1946), the American Museum of Natural History expedition in 1941 on the diesel schooner ‘Askoy’ (Nichols and Murphy 1944), and a joint U.S. Navy – Smithsonian Tropical Research expedition to Malpelo Island in 1972 (Findley 1975; Graham 1975; McCosker and Rosenblatt 1975). More recent foreign surveys include those made by the Japan International Technical Cooperation Agency (JICA, 1981), the exploratory fisheries of the Spanish Cooperation Agency (Balguerías and Rodríguez 1983), and the Norwegian Exploration Agency (NORAD) (Stromme and Sætersdal 1988).

Colombian scientists continued to build knowledge about Colombian Pacific fishes throughout the 1970s and 1980s. Academic institutions published species lists, including those by Sterling (1975, 1976, 1977), Gómez and Díaz (1979), and Rubio (1982, 1984, 1987, 1988), which served as the foundations for the current effort. Significant attention has been given to the fish fauna of Gorgona Island, a national natural park with a variety of habitats, including unique coral reefs (Rubio et al. 1987; Rubio 1990; Franke and Acero 1991, 1992a, 1992b, 1993, 1995, 1996; Acero and Franke 1993, 1995, 2001; Zapata 2001a, 2001b; Rojas and Zapata 2006; Payán et al. 2010; Alzate et al. 2012). The now dismantled Instituto Nacional para la Pesca y Acuicultura also played an important role as a prospector of the region’s existing fishery resources (Zapata et al. 1999a, 1999b; Beltrán-León and Ríos-Herrera 2000), contributing to increased regional knowledge. Recent work includes new species descriptions (e.g., Acero and Betancur 2002; Tavera 2021; Tavera et al. 2021; Mejía-Falla et al. 2025), new records (e.g., Beltrán-León and Rubio 1994; Zapata and Morales 1995; Acevedo et al. 1998; Gómez et al. 2001; Puentes et al. 2001; Pedraza et al. 2002; Rubio et al. 2005a, 2005b; Mejía-Falla and Navia 2009; Beltrán-León et al. 2016; Rojas-Vélez and Tavera 2017; Tavera and Rojas-Vélez 2017; Beltrán-León and Ríos 2018), and updated species lists (e.g., Beltrán-León 2007; Mejía-Falla et al. 2007; Puentes et al. 2007; Tobón-López et al. 2008; Navia et al. 2009, 2016; Zapata and Usma 2013; Mejía-Falla and Navia 2019), among others.

For more than three decades, the late Professor Efraín A. Rubio (from Universidad del Valle) worked determinedly to compile a series of annotated species lists of the Colombian Pacific fish fauna. In this daunting endeavor, he included species which occurred in neighboring countries, without vouchers, inferring that they should also be present in Colombia. While his lists have long served as the basis for a full inventory, it is now necessary to examine the evidence supporting the occurrence of species in Colombian Pacific jurisdictional waters either as specimens in collections or photographic records. This paper aims to produce a depurated species list of Colombian Pacific fishes using the following sources: material deposited in Colombian museums, ROV photographs, published books or papers of new records or additions to Colombian Pacific fish fauna, species that have been described based on material collected in Colombia but whose material is stored in collections outside the country, and lastly two published lists of Colombian material deposited in US collections, personally reviewed by their authors, some of whom are co-authors of this work. All this information will serve as a first step towards completing a verified species inventory. The list presented here, rather than serving as an addition to the existing taxonomic records, seeks to purge unsupported records of all species that have uncertain taxonomic identification, are not backed by vouchered specimens, or from recently published records based on specimens in reference collections. We acknowledge that this list may fall short on numbers and be overly stringent; however, every species included can be considered a validated record, in the hope of contributing to the consolidation of a reliable taxonomic list to which more species can be added.

### Colombian Pacific

**Figure 1.**
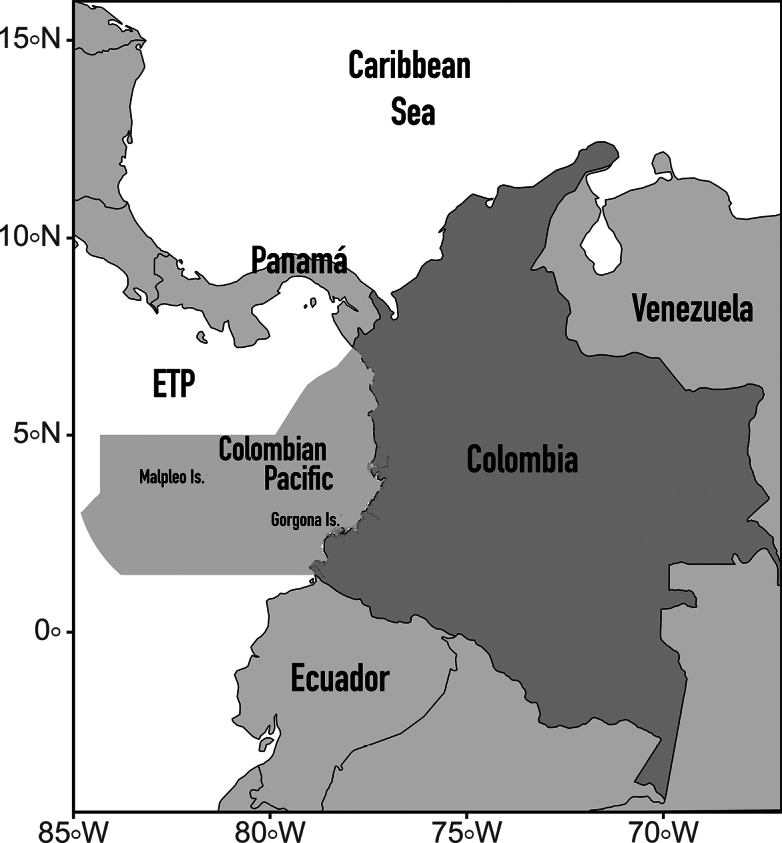
Geographic map of Colombia. The area of the Colombian Pacific Ocean is highlighted in gray.

The Colombian Pacific Ocean covers ca 350,000 km^2^ and encompasses the western coast of northern South America from 7.2°N on the Colombia–Panama border to 1.4°N at the border with Ecuador, and includes the Gorgona and Malpelo Islands, as well as a large expanse of open ocean (Fig. 1). The Colombian marine territory in the Pacific represents 16.4% of the national territory and ~40% of the country’s maritime area. The Colombian Pacific coast extends more than 1500 km with a predominantly north-south orientation and has a highly varied topography produced by the collision of the Nazca and South American tectonic plates (Suárez-Rodríguez 2007; Restrepo and López 2008; Correa and Morton 2010). The coast can be divided into two different regions based on its geomorphology: the northern extends from the border with Panama south to Cabo Corrientes. This ~375-km strip is demarcated by the Serranía del Baudó coastal mountain range, which runs in a north-south direction, with gentle eastern slopes and steep western slopes which form small beaches and rocky cliffs (Galvis and Mojica 1993), as well as small mangrove patches that develop around river mouths (Castellanos-Galindo et al. 2021). This area also contains the northern coastal reef formations of the Gulf of Cupica and Ensenada de Utría (Zapata and Vargas-Ángel 2003). On the other hand, the southern region has low relief and sedimentary environments, with numerous nearshore islets that extend from south of Cabo Corrientes to the border with Ecuador (Martínez et al. 1995). These low coastal plains are made up of sediments from the late Quaternary, in which mangroves and freshwater sources (San Juan, Patía, and Mira rivers) are prevalent, depositing a great deal of sediment, in the form of coastal fans and sand bars (Galvis and Mojica 1993). This area contains the most structurally complex and thus better-developed mangrove forest in the Colombian Pacific. This regionalization is also supported by the ocean bathymetry adjacent to the coast, the north has a narrow continental shelf (the 200-m isobath is ~15 km from the coast), whereas in the south, it is approximately four times wider. The entire Colombian Pacific lies within the Panama Bight, one of eight ETP ecoregions (Spalding et al. 2007), encompassing the area between the Azuero Peninsula (Panama) and the Ancón de Sardinas (Ecuador).

The Colombian Pacific has relatively warm surface waters (25–26 °C), and variable salinities (20–35 ‰) depending on the proximity to the coast (Cantera and Contreras 1993; Herrera-Carmona et al. 2022). It is located in a region of low pressure, known as the equatorial concavity, where the trade winds of each hemisphere converge forming the Intertropical Convergence Zone, characterized by the presence of variable winds in addition to high rainfall (Poveda et al. 2006). The variability of geomorphological, oceanographic, and climatic conditions makes this a highly biologically diverse region.

## Methods

The majority of the species included in this list came from published inventories and a reappraisal of specimens deposited in registered Colombian ichthyological collections, each of which is associated with a voucher specimen or a photograph with georeferenced data (Rubio 1982, 1984, 1987, 1988; Allen and Robertson 1994; Béarez 1996; Beltrán-León and Ríos-Herrera 2000; Chirichigno and Cornejo 2001; Rojas and Zapata 2006; Mejía-Falla et al. 2007; Zapata and Usma 2013; Robertson and Allen 2024).

We based our list on confirmed records available from the main recognized Colombian marine ichthyological collections: Universidad del Valle (CIR-UV), Instituto de Investigaciones Marinas y Costeras -INVEMAR- (INV PEC), Parque Nacional Natural Gorgona (PE and MMG), and Instituto de Ciencias Naturales de la Universidad Nacional de Colombia (ICN-MHN). The exceptions to the deposited records are as follows: 1) photographs for large mobile or rare species, only if the original source was available with georeferenced data. 2) If a species was described based on Colombian specimens, whether it is considered valid or a synonym by the Catalog of Fishes. 3) Species listed in Castellanos-Galindo et al. (2006b, 2006c) since the authors personally reviewed the Colombian material deposited at several international museums. 4) Species-specific published records with photographs included but the specimen was not deposited in a fish collection. 5) ROV photographs from deep sea expeditions. Problematic taxa and those whose images are insufficient to validate identification at the species level were not included. In cases where the identity of the species was questionable or its taxonomy complex, physical examinations of the individuals were carried out. In cases where the batches were too numerous to examine all of them, a few individuals were selected from the batch and identified. We avoided any data aggregators, and other sources that may recycle information, which would prevent this depuration from being feasible. Additionally, we compiled a list of ETP species fish larvae for which the adults have never been found or collected in Colombia and species that have been listed in either north (Panama) or south (Ecuador) but have never been collected or have no traceable records in Colombian territorial waters.

Nomenclature and name validity were based on Eschmeyer’s Catalog of Fishes (Fricke et al. 2024b). The checklist is arranged phylogenetically at the ordinal and familial levels following Betancur-R et al. (2017) for ray-finned fishes (Actinopterygii). Genera and species within families are arranged in alphabetical order. For Elasmobranchii, Nelson et al. (2016) are followed and, when indicated, Last et al. (2016a).

In addition to establishing the total taxa, we assigned the species to different ecological categories according to Froese and Pauly (2024) and Robertson and Allen (2024). These categories were based on 1) vertical position in the water column (benthic if completely associated with the bottom; bentho-pelagic if closely associated with the bottom but not permanently in contact with it; and pelagic if mostly associated with the water column with little or no contact with the bottom). 2) pelagic species were subdivided according to the proximity to shore into coastal, coastal-oceanic, and oceanic. 3) benthic and benthopelagic species were divided according to preferred habitat/substrate type: hard bottom, if they inhabit predominantly any type of hard substratum such as reefs, soft bottom, if they inhabit mud, sand, rubble, or similar substrates, and mixed, when there is no apparent preference for either habitat/substrate type. 4) A category related to exploitation was assigned to those species used for commercial and subsistence purposes (commercial and non-commercial). Considering that at the date of finishing this inventory (first semester of 2025), there is a national debate in Colombia about the status of cartilaginous fishes as a source of food and income, we decided to refrain from categorizing any of these species as commercial or non-commercial until the dispute is settled. 5) Depth (shallow, species found within the first 50 m; intermediate, species found at > 50–200 m; and deep, species found at depths > 200 m. 6) Finally the International Union for Conservation of Nature regional threat categories, based on the red list of marine fishes of Colombia (Chasqui et al. 2017).

## Results and discussion

In the process of compiling this checklist, several challenges arose when verifying the presence of species. Therefore, far from being complete, this list underwent a rigorous validation process, with each record confirmed by either one author, or, in many cases, by all authors through validation of vouchered specimens. As far as we can tell, the list is complete with respect to all the verifiable available sources in Colombia by this manuscript’s submission date.

Overall, 727 marine fish species (Table 1) were reliably verified to be present in the Colombian Pacific (https://doi.org/10.15472/kmsezq); this represents ~50% of the 1468 confirmed marine, shallow water species found in the ETP (Robertson and Allen 2024); however, the latter does not include deep-sea fish, which makes comparison complicated. Our list is significantly shorter than that presented by Rubio (1987), who reported 964 species for the Colombian Pacific but is close to Robertson and Allen’s (2024) database, according to which 857 species are found in our region. Our revised list (Table 1) included three classes, of which 48 orders, 122 families, 375 genera, and 663 species correspond to fin-rayed fishes: 11 orders, 27 families, 36 genera, and 64 species to cartilaginous fishes, and one order, one family, one genus, and one species of hagfish. Of the 727 species, 35 (in 20 families) are new records for Colombia, 15 species are Colombian endemics. Two additional tables are also included, first a list of 59 species reported from tropical waters both north and south of the Colombian borders but lack confirmed records within Colombian waters (Table 2), and a list of 77 species that have been recorded only in their larval stages (Table 3). ROV records of the species included can be found in https://ipt.biodiversidad.co/sibm/resource?r=seamountsexpedition_2021 as well as in the final report of the ARGO expedition (Parques Nacionales Naturales de Colombia 2022). The photographs owned by Parques Nacionales Naturales de Colombia may be requested from the authors or directly from Parques Nacionales Naturales de Colombia.

**Table 1.** Commented list of Colombian Pacific marine fishes. Acronyms used are as follows: AMNH = American Museum of Natural History New York, USA; ANSP = Academy of Natural Sciences of Philadelphia, Philadelphia, USA; BMNH: British Museum of Natural History, London, England; CAS = California Academy of Science, California, USA; CIRUV = Colección Ictiológica de Referencia Universidad del Valle, Cali, Colombia; ICNMHN = Instituto de Ciencias Naturales Museo de Historia Natural, Bogotá, Colombia; INV PEC = Museo de Historia Natural Marina de Colombia, INVEMAR, Santa Marta, Colombia; IMCN = Colección de Referencia Museo Departamental de Ciencias Naturales-INCIVA, Cali, Colombia; GCRL = Gulf Coast Research Laboratory, Ocean Springs, Mississippi, USA; LACM = Los Angeles County Museum, Los Angeles, USA; MCZ = Museum of Comparative Zoology, Cambridge, USA; PE and MMG = Museo Marino Peces de Gorgona, Colombia; MNHN: Museum national d’histoire naturelle, Paris, France; SIO = Scripps Institution of Oceanography, San Diego, USA; SU = Stanford University, USA (now in CAS); USNM = National Museum of Natural History Smithsonian, Washington D.C., USA; ZMUC = Zoological Museum University of Copenhagen, Copenhagen, Denmark.

Taxa	Records	Comments	Only Malpelo Island	Endemic	New records	References
** Myxini **						
** Myxiniformes **						
** Myxinidae **						
*Myxine circifrons* Garman, 1899	CIRUV 95002					
** Chondrichthyes **						
** Euselachii **						
** Elasmobranchii **						
**Selachii**						
** Heterodontiformes **						
** Heterodontidae **						
*Heterodontus cf. francisci* (Girard, 1855)	CIRUV 90005; 78082	This material was identified as *H. cf. francisci* pending on going molecular and morphological studies. Previous specimens indentified as *H. quoyi* associated with CIRUV are included in this taxon				
*Heterodontus mexicanus* Taylor & Castro-Aguirre, 1972	CIRUV 23113					
** Orectolobiformes **						
** Ginglymostomatidae **						
*Ginglymostoma unami* Del Moral-Flores, Ramírez-Antonio, Angulo & Pérez-Ponce de León, 2015	Underwater photography at Gorgona Island					Rubio 1986; Rubio et al. 1987a; Acero and Franke 2001
** Rhincodontidae **						
*Rhincodon typus* Smith, 1828	PE 88117					
** Lamniformes **						
** Alopiidae **						
*Alopias pelagicus* Nakamura, 1935	CIRUV 006020					
*Alopias superciliosus* Lowe, 1841	CIRUV 010176; 010177					
** Lamnidae **						
*Odontaspis ferox* (Risso, 1810)	Underwater photography at Malpelo Island		**x**			Bessudo and Lefevre 2017
** Carcharhiniformes **						
** Carcharhinidae **						
*Carcharhinus albimarginatus* (Rüppell, 1837)	MMG 91126;PE 88048;PE 92093; CIRUV 023017					
*Carcharhinus altimus* (Springer, 1950)	PE 89097, female mandible					Franke and Acero 1991
*Carcharhinus cerdale* Gilbert, 1898	CIRUV 80080; 81029; 78038; 79020; 75009; 78039; 88162; 81030; 058-002	This species was previously identified as *C. porosus* (Ranzani, 1839) being the late only valid for the Atlantic basin				
*Carcharhinus falciformis* (Bibron, 1839)	CIRUV 010065; 010092; 010094; Fotos Malpelo					
*Carcharhinus galapagensis* (Snodgrass & Heller, 1905)	Underwater photography at Malpelo Island					Gómez and Díaz 1979; Rubio 1986; Rubio et al. 1987a
*Carcharhinus leucas* (Valenciennes, 1839)	CIRUV 024069					
*Carcharhinus limbatus* (Valenciennes, 1839)	PE 91109; PE 91099; PE 89037; CIRUV 010183; 023079					
*Carcharhinus longimanus* (Poey, 1861)	Underwater photography at Gorgona Island					Bessudo and Lefevre 2017
*Nasolamia velox* (Gilbert, 1898)	PE 87071; PE 90109; PE 90075; CIRUV 010101; 010105; 023114; 023089; 023363					
*Negaprion fronto* (Jordan & Gilbert, 1882)	PE 88103, female mandible					Franke and Acero 1991
*Prionace glauca* (Linnaeus, 1758)	ICN-MHN-477					
*Rhizoprionodon longurio* (Jordan & Gilbert, 1882)	PE 88005; PE 90149; PE 91010; CIRUV 023010; 023081; 023136; 023139; 023145; 023151; 023161; 023373; 023494; 023499					
*Triaenodon obesus* (Rüppell, 1837)	PE 87078; CIRUV 023020					
** Galeocerdonidae **						
*Galeocerdo cuvier* (Péron & Lesueur, 1822)	PE 91008	This species has recently been placed in its own family sister to the remaining carcharhinids. Spelled Galeocerdidae in Herman and Van den Eeckhaut (2010), but also as Galeocerdonidae in Fricke et al. (2024b)				
** Pentanchidae **						
*Apristurus* sp.	INV PEC8708	This genus is included in the family Scyliorhinidae in Nelson et al. (2016). *Apristurus brunneus* (Gilbert, 1892) and *A. nasutus* de Buen, 1959, were reported by Rubio (1987) but the vouchered specimens were not found. Bessudo et al. (2021) reported *A. kampae* Taylor, 1972, at Malpelo Island based on an ROV photo; however, species identification in this genus is not trivial. Hence, we decided not to go as far as species on our list				Bessudo et al. 2021
** Sphyrnidae **						
*Sphyrna corona* Springer, 1940	CIRUV 81215; 74006					
*Sphyrna lewini* (Griffith & Smith, 1834)	CIRUV 78165; 81214; 010178; 021155; 023409; 023410					
*Sphyrna media* Springer, 1940	CIRUV 81255; 021335					
*Sphyrna mokarran* (Rüppell, 1837)	Underwater photography at Malpelo Island					Rubio 1986; Rubio et al. 1987a; Mejia-Falla and Navia 2019
*Sphyrna vespertina* Springer, 1940	CIRUV 78166; 78167; 79115; 81216; 90047; 058-001; 017015; 017018; 17026; 17027; 021256; 023541; 023542;	Previously considered a subspecies of the western Atlantic *Sphyrna tiburo* Linnaeus, 1758. The TEP species is genetically and morphologically distinct (Aroca et al. 2022)				
** Triakidae **						
*Mustelus dorsalis* Gill, 1864	CIRUV 006018; 017039; 023365; 023394					
*Mustelus henlei* (Gill, 1863)	CIRUV 74003; 78179; 75017; 19068; 023038; 023094; 023097; 023540					
*Mustelus lunulatus* Jordan & Gilbert, 1882	PE 91096; PE 92047; PE 92029; CIRUV 010163; 023086; 023099; 023362; 023364; 023470					
*Mustelus whitneyi* Chirichigno F., 1973	CIRUV 017048				**x**	
** Hexanchiformes **						
** Hexanchidae **						
*Notorynchus cepedianus* (Péron, 1807)	PE 88075; PE 90151; CIRUV 010160; 023359; 023369					
** Squaliformes **						
** Etmopteridae **						
*Etmopterus* sp.	Underwater photography at Malpelo Island	Bessudo et al. (2021) identified *Etmopterus* specimens as cf *granulosus* (Günther, 1880), a Southern Hemisphere species, based on an ROV photo at Malpelo Island. We consider species identification of this dwarf shark genus not trivial; the picture in the publication is not conclusive for species- level identification	**x**			Bessudo et al. 2021
*Centroscyllium nigrum* Garman, 1899	CIRUV 95001					
** Echinorhiniformes **						
** Echinorhinidae **						
*Echinorhinus cookei* Pietschmann, 1928	Photograph in Navia et al. (2023) and ROV underwater photography at deep mountains at Malpelo-Yuruparí					Navia et al. 2023
** Squatiniformes **						
** Squatinidae **						
*Squatina californica* Ayres, 1859	CIRUV 90012; 010165; 023356; 023360; 023368; PE 90089	Material held at CIRUV was erroneously identified as the Perú-Chile *Squatina armata* (Philippi, 1887). The latter name has been widely and uncritically used for Colombian Pacific angel sharks				
**Batomorphi**						
** Torpediniformes **						
** Narcinidae **						
*Narcine entemedor* Jordan & Starks, 1895	CIRUV 86024; 007001; 010007; 010095; 010175; 021096; 023395					
*Narcine leoparda* Carvalho, 2001	CIRUV 88008; 88033; 75050; 05179; 005-233; 88031; 88144; 78177; 81237; 80405; 78178; 79126; 006-001; 85062; 81238; 005-233; 006-001; 010106; 018008					
** Torpedinidae **						
*Tetronarce tremens* (de Buen, 1959)	ROV underwater photography at deep mountains at Malpelo-Yuruparí		**x**			
** Rhinopristiformes **						
** Pristidae **						
*Pristis pristis* (Linnaeus, 1758)	CIRUV 88228					
** Rhinobatidae **		According to Last et al. (2016b), amphi-American members of *Rhinobatos* were assigned to the genus *Pseudobatos*				Last et al. 2016b
*Pseudobatos leucorhynchus* (Günther, 1867)	CIRUV 81157; 88220; 88007; 78120; 005-232; 81158; 78050; 88032; 79085; 80289; 005144; 005018; 008118; 005178; 005096					
*Pseudobatos prahli* (Acero P. & Franke, 1995)	ICN-MHN 4049					Acero and Franke 1995; Payán et al. 2010
** Trygonorrhinidae **						
*Zapteryx xyster* Jordan & Evermann, 1896	CIRUV 85074; 90006; 010063; 010100; 010180; 017073; 021048; 021224; 023325; 023326; 023376; 023400; 023403; 023404					
** Rajiformes **						
** Arhynchobatidae **						
*Bathyraja* sp.	ROV underwater photography INVEMAR-UNIVALLE	Bessudo et al. (2021) identified *Bathyraja*, based on a photo using ROV at Malpelo Island, as *B. spinosissima* (Beebe & Tee-Van, 1941). While we consider this identification possible, this genus is one of the most speciose groups of rays in the world, and identifying any species is not a trivial task. Physical examination of specimens is necessary to identify to species correctly	**x**			
** Rajidae **						
*Amblyraja hyperborea* (Collett, 1879)	ROV underwater photography INVEMAR-UNIVALLE		**x**			
*Rostroraja equatorialis* (Jordan & Bollman, 1890)	CIRUV 017065; 018251					
*Rostroraja velezi* (Chirichigno F., 1973)	CIRUV 95011; 023357; 023370; 023397; 023398					
** Myliobatiformes **						
** Aetobatidae **						
*Aetobatus laticeps* (Gill, 1865)	CIRUV 010098; 023525	Victor et al. (2024), based on a personal comment by Diana Pazmino, stated that molecular data supports that *Aetobatus ocellatus* (Kuhl, 1823) is the species found in the Galapagos, and extend this finding to Cocos and Malpelo, based on coloration. We have evidence of significant color variation within the *Aetobatus* species. Therefore, until a specific study is published, we prefer to be cautious and leave it as *A. laticeps* pending further detailed examination of the Malpelo specimens				
** Dasyatidae **						
*Hypanus dipterurus* (Jordan & Gilbert, 1880)	Underwater photography at Gorgona Island					Zapata et al. 1999b
*Hypanus longus* (Garman, 1880)	PE 91095; PE 91110; PE 91068					
*Hypanus rubioi* Mejía-Falla, Navia, Cardeñosa & Tavera, 2025	CIRUV 021336, CIRUV 024008, CIRUV 024073-75				**x**	
** Gymnuridae **						
*Gymnura crebripunctata* (Peters, 1869)	CIRUV 006002; 005180; 78079	Material held at CIRUV was erroneously identified as the North American butterfly ray *Gymnura marmorata* (Cooper, 1864). This name has been and is still widely and uncritically used for Colombian Pacific butterfly rays. Ongoing molecular studies on the Colombian specimens are being carried out				
** Mobulidae **						
*Mobula birostris* (Walbaum, 1792)	Underwater photographs at Gorgona and Malpelo Island					Rubio 1986; Rubio et al. 1987a; Acero and Franke 1995, 2001
*Mobula munkiana* Notarbartolo-di-Sciara, 1987	PE 89004					
*Mobula thurstoni* (Lloyd, 1908)	CIRUV 023401; 023402; 023324; 023480					
** Potamotrygonidae **						
*Styracura pacifica* (Beebe & Tee-Van, 1941)	Photograph in Scheel-Dalmau et al. (2020)					Scheel-Dalmau et al. 2020
** Rhinopteridae **						
*Rhinoptera steindachneri* Evermann & Jenkins, 1891	PE 92049; CIRUV 023469; 023399; 023406;					
** Urotrygonidae **						
*Urobatis pardalis* Del Moral-Flores, Angulo, López & Bussing, 2015	CIRUV 87022	According to Del Moral-Flores et al. (2015), *Urobatis halleri* (Cooper, 1863), previously confused with *U. pardalis*, extends from Oregón, USA, to southern México				Del Moral-Flores et al. 2015
*Urobatis tumbesensis* (Chirichigno F. & McEachran, 1979)	CIRUV 006-0063 007-0094					Mejía-Falla and Navia 2009
*Urotrygon aspidura* (Jordan & Gilbert, 1882)	CIRUV 006-011; 006-012; 00416; 00427; 00414; 00417; 00426; 00480;U113;U20;U49;U22;U38;U119;U62;U25;U15; 006-0042; 005-145					
*Urotrygon munda* Gill, 1863	CIRUV 81241; 88068					
*Urotrygon reticulata* Miyake & McEachran, 1988	CIRUV 018062					
*Urotrygon rogersi* (Jordan & Starks, 1895)	CIRUV 252; 008-114; 007-116; 007-0002; 006-0043; 059;U98;U113;U114;U43;U64;U97;U9;U12;U4;U92;U29;U93;U95;U54;U108; 006-0040; 007-0055; 78185; 86001; 88143; 78185; 88029; 88009; 005-035; 005-051; 009-120; 006-0041; 005-0197;U111;U123;U92; 78186; 80505; 006-0014; 005052; 008114; 009096; 006-013; 88030; 8503; 0050010; 003-006; 0040015; 004029; 0040094; 0040079; 004003; 006-096; 80504	*Urotrygon caudispinosus* Hildebrand, 1946, is a synonym of *U. munda* Gill, 1863. Material from Colombia wrongly reported as that species, is now reidentified as *U. rogersi* based on material deposited at CIRUV				
*Urotrygon simulatrix* Miyake & McEachran, 1988	INV PEC 7636; 7694					
** Actinopterygii **						
** Actinopteri **						
** Neopterygii **						
** Teleostei **						
** Elopomorpha **						
** Elopiformes **						
** Elopidae **						
*Elops affinis* Regan, 1909	PE 87032; CIRUV 010069; 023031					
** Megalopidae **						
*Megalops atlanticus* Valenciennes, 1847	Catch pictures at Bahia Malaga, Valle del Cauca, and sport fishing at Chocó.	Invasive Atlantic species that began crossing to the Eastern Pacific through the Panama Canal ~100 yrs ago. Nowadays it is widely caught from all the Colombian Pacific shores				
** Albuliformes **						
** Albulidae **						
*Albula esuncula* (Garman, 1899)	CIRUV 80001; 80010; 86008; 81060; 80002					
*Albula pacifica* (Beebe, 1942)	CIRUV 006019					
** Notacanthiformes **						
** Halosauridae **						
*Aldrovandia phalacra* (Vaillant, 1888)	ROV underwater photography INVEMAR-UNIVALLE		**x**		**x**	
*Halosaurus radiatus* Garman, 1899	INV PEC6842; 6843; 6844; 8710; 8711; 8712; 8713; 8714; 8715				**x**	
** Anguilliformes **						
** Congridae **						
*Ariosoma gilberti* (Ogilby, 1898)	Holotype MCZ 28425	*Atopichthys acus* Garman, 1899 (Holotype, unique: MCZ 28425), synonym of *Ariosoma gilberti*, collected off Chocó, Colombia				
*Bathycongrus macrurus* (Gilbert, 1891)	CIRUV 90056; 0010087					
*Gnathophis cinctus* (Garman, 1899)	CIRUV 002-0023; 023548; 023552					
*Heteroconger klausewitzi* (Eibl-Eibesfeldt & Köster, 1983)	Underwater photography at Gorgona Island					Palacios et al. 2022
*Heteroconger pellegrini* Castle, 1999	CIRUV 019057; 021341					Palacios et al. 2022
*Japonoconger proriger* (Gilbert, 1891)	CIRUV 023554					
*Rhynchoconger nitens* (Jordan & Bollman, 1890)	ICN-MHN 4530; CIRUV 002-0021; 023553					
*Xenomystax atrarius* Gilbert, 1891	CIRUV 95007a; 023550					
** Heterenchelyidae **						
*Pythonichthys asodes* Rosenblatt & Rubinoff, 1972	CIRUV 80121; 89015; 021195; 023545; 023546; 023547; 023549					
** Moringuidae **						
*Neoconger vermiformis* Gilbert, 1890	ICN MHN 4521; CIRUV 024064					
** Muraenesocidae **						
*Cynoponticus coniceps* (Jordan & Gilbert, 1882)	CIRUV 90056; 0010087					
** Muraenidae **						
*Anarchias galapagensis* (Seale, 1940)	INV PEC 6200					
*Echidna nebulosa* (Ahl, 1789)	CIRUV 80242; 018081; 023518					
*Echidna nocturna* (Cope, 1872)	CIRUV 87001; 8243; 004070; 005076; 78092; 78091; 78093b; 78093a					
*Enchelycore octaviana* (Myers & Wade, 1941)	CIRUV 005150; 005160					
*Gymnomuraena zebra* (Shaw, 1797)	PE 89047					
*Gymnothorax angusticeps* (Hildebrand & Barton, 1949)	ROV underwater photography at deep mountains at Malpelo-Yuruparí		**x**			
*Gymnothorax buroensis* (Bleeker, 1857)	Underwater photography at Gorgona Island				**x**	
*Gymnothorax castaneus* (Jordan & Gilbert, 1883)	CIRUV 78190; 80245					
*Gymnothorax dovii* (Günther, 1870)	PE 89062; CIRUV 78191; 023066					
*Gymnothorax equatorialis* (Hildebrand, 1946)	CIRUV 81131; 75034; 001-0010					
*Gymnothorax flavimarginatus* (Rüppell, 1830)	Underwater photographs at Gorgona and Malpelo Island					Zapata and Morales 1997; Muñoz and Zapata 2013
*Gymnothorax panamensis* (Steindachner, 1876)	CIRUV 006006; 005151; 005156; 005162; 83001					
*Gymnothorax phalarus* Bussing, 1998	CIRUV 78190; 78093					
*Gymnothorax undulatus* (Lacepède, 1803)	CIRUV 90048; 80246; 006-005					
*Muraena argus* (Steindachner, 1870)	CIRUV 020017; 020021; 023029; 023380; 023381					
*Muraena clepsydra* Gilbert, 1898	CIRUV 75035; 80247; 005155; 05158; 005146; 86002					
*Muraena lentiginosa* Jenyns, 1842	CIRUV 78097; 85075; 005147; 005149; 005159					
*Scuticaria tigrina* (Lesson, 1828)	Underwater photography FZ					
*Uropterygius macrocephalus* (Bleeker, 1864)	PE 87043; CIRUV 010031					
*Uropterygius versutus* Bussing, 1991	CIRUV 008-0076					
** Myrocongridae **						
*Myroconger nigrodentatus* Castle & Béarez, 1995	ROV underwater photography at deep mountains at Malpelo-Yuruparí		**x**		**x**	
** Nemichthyidae **						
*Nemichthys scolopaceus* Richardson, 1848	CIRUV 75036; 95017					
** Nettastomatidae **						
*Hoplunnis pacifica* Lane & Stewart, 1968	CIRUV 78089; 91010					
** Ophichthidae **						
*Aplatophis zorro* McCosker & Robertson, 2001	CIRUV 079001; 023447					
*Apterichtus equatorialis* (Myers & Wade, 1941)	CIRUV 80086; 012129; 023559				**x**	
*Echiophis brunneus* (Castro-Aguirre & Suárez de los Cobos, 1983)	CIRUV 80253; 081138					
*Gordiichthys combibus* McCosker & Lavenberg, 2001	Holotype ANSP 130223; Paratypes ANSP 138561; CAS 208467; USNM 356856	Colombian endemic holotype: ANSP 130223. Paratypes: ANSP 138561 (2); CAS 208467; USNM 356856		**x**		
*Myrichthys xysturus* (Jordan & Gilbert, 1882)	CIRUV 79071; 81135; 90004; 010049; 011040					
*Myrophis vafer* Jordan & Gilbert, 1883	ICN-MHN 4523; CIRUV 75011; 021099					
*Ophichthus apachus* McCosker & Rosenblatt, 1998	Paratype CAS-ICH 97856	Paratype from Utría				
*Ophichthus frontalis* Garman, 1899	CIRUV 021124; 023383					
*Ophichthus remiger* (Valenciennes, 1837)	CIRUV 76021; 78100; 79030; 80250; 91038; 92003; 023229					
*Ophichthus triserialis* (Kaup, 1856)	CIRUV 97002; 006011; 010023; 010024; 011015					
*Ophichthus zophochir* Jordan & Gilbert, 1882	ICN-MHN 4524; CIRUV 78102; 80251; 80252; 88012; 0030043; 0070030; 010058					
*Phaenomonas pinnata* Myers & Wade, 1941	Holotype LACM 21560 [ex AHF 13]; Paratypes LACM 21705	Described based on material collected at Utria, Chocó holotype: LACM 21560 [ex AHF 13]. Paratypes: LACM 21705				
*Pisodonophis daspilotus* Gilbert, 1898	CIRUV 003-0042; 018089					
*Quassiremus evionthas* (Jordan & Bollman, 1890)	CIRUV 023182				**x**	
** Synaphobranchidae **						
*Ilyophis* sp.	ROV underwater photography INVEMAR-UNIVALLE	ROV images of this deep-sea eel are difficult to identify without physically examining the specimens. However, it is a valid record and adds to our species richness	**x**			
** Otomorpha **						
** Clupeiformes **						
** Alosidae **						
*Sardinops sagax* (Jenyns, 1842)	CIRUV 90003; 023131					
** Dorosomatidae **						
*Harengula thrissina* (Jordan & Gilbert, 1882)	ICN-MHN 4540; CIRUV 0010003; 80109					
*Lile stolifera* (Jordan & Gilbert, 1882)	CIRUV 003-0027; 80112; 85022; 80115					
*Opisthonema bulleri* (Regan, 1904)	CIRUV 81064; 0010002					
*Opisthonema libertate* (Günther, 1867)	CIRUV 79029; 010133; 016038; 021153; 022003					
*Opisthonema medirastre* Berry & Barrett, 1963	CIRUV 81071; 88132; 021226; 023303					
** Engraulidae **						
*Anchoa argentivittata* (Regan, 1904)	CIRUV 80142; 79041; 75024; 85019; 80143; 80144; 79040					
*Anchoa curta* (Jordan & Gilbert, 1882)	CIRUV 80135; 80136					
*Anchoa eigenmannia (*Meek & Hildebrand, 1923)	CIRUV 005-0191					
*Anchoa ischana* (Jordan & Gilbert, 1882)	CIRUV 80137; 90022; 0040163; 80138; 81080					
*Anchoa lucida* (Jordan & Gilbert, 1882)	CIRUV 007-0039; 03-0046; 002-015; 0010030; 79037; 80139; 81082; 80140; 002-013					
*Anchoa nasus* (Kner & Steindachner, 1867)	CIRUV 80142; 79041; 75024; 85019; 80143; 80144; 79040					
*Anchoa panamensis* (Steindachner, 1876)	CIRUV 89028; 88199; 80147; 005-065; 003-0030; 80146; 80145; 75025; 009-91; 81084					
*Anchoa spinifer* (Valenciennes, 1848)	CIRUV 00502; 78060; 94015; 001007; 005003; 0040033; 85018; 88129; 79043; 0040083					
*Anchoa starksi* (Gilbert & Pierson, 1898)	CIRUV 02-0014					
*Anchoa walkeri* Baldwin & Chang, 1970	CIRUV 005-066					
*Anchovia macrolepidota* (Kner, 1863)	ICN-MHN 4536; CIRUV 89040; 80150; 88191; 79038; 88118; 81083; 88206; 86027; 89067; 70004; 80141; 75026					
*Cetengraulis mysticetus* (Günther, 1867)	ICN-MHN 4537; CIRUV 016008; 88125; 85020; 85021; 016014; 003-0026; 005-030; 009-065; 008-119; 0040082					
** Pristigasteridae **						
*Ilisha fuerthii* (Steindachner, 1875)	CIRUV 81063; 81061; 81064; 88130; 79027					
*Neoopisthopterus tropicus* (Hildebrand, 1946)	ICN-MHN 4538; CIRUV 72005; 81068					
*Odontognathus panamensis* (Steindachner, 1876)	CIRUV 023239					
*Opisthopterus dovii* (Günther, 1868)	CIRUV 78051; 80118; 81072; 81073; 86025; 88063; 90021; 001019; 0010057; 0010060; 005-092; 023185; 023242; 023293; 023298					
*Opisthopterus effulgens* (Regan, 1903)	ICN-MHN 4539; CIRUV 005-079; 016007; 016037			**x**		
*Opisthopterus equatorialis* Hildebrand, 1946	CIRUV 72006; 016025; 016024; 005-041; 0070028; 023092; 023232; 023299					
*Pliosteostoma lutipinnis* (Jordan & Gilbert, 1882)	CIRUV 72006; 016025; 016024; 005-041					
** Gonorynchiformes **						
** Chanidae **						
*Chanos chanos* (Fabricius, 1775)	Underwater photographs at Gorgona and Malpelo Island					Franke and Acero 1992
** Siluriformes **						
** Ariidae **						
*Ariopsis simonsi* (Starks, 1906)	CIRUV 90032; 005-050					
*Bagre panamensis* (Gill, 1863)	CIRUV 0010018; 78003; 81036; 81000; 81056; 0010075; 80048; 004-0103; 016; 01604; 900310					
*Bagre pinnimaculatus* (Steindachner, 1876)	CIRUV 76022; 81055; 88071; 88164; 0010031; 78002; 80001; 80010; 79003; 8004; 81001					
*Cathorops dasycephalus* (Günther, 1864)	USNM 214929; 214930	AAP personally revised those Colombian vouchers at USNM Washington				Rojas and Zapata 2006
*Cathorops manglarensis* Marceniuk, 2007	CIRUV 016026; 021123					
*Cathorops multiradiatus* (Günther, 1864)	CIRUV 005153					
*Cathorops* sp.	CIRUV 021190			**x**	**x**	
*Cathorops steindachneri* (Gilbert & Starks, 1904)	CIRUV 016042; 021248					
*Notarius armbrusteri* Betancur-R. & Acero P., 2006	INV PEC 6677; 6678; 6719					
*Notarius kessleri* (Steindachner, 1876)	INV PEC 6717; 6785					
*Notarius* sp.	CIRUV 024003			**x**	**x**	
*Notarius troschelii* (Gill, 1863)	CIRUV 016027; 021157					
*Occidentarius platypogon* (Günther, 1864)	CIRUV 90061					
*Sciades dowii* (Gill, 1863)	CIRUV 011070; 016028					
** Euteleosteomorpha **						
** Argentiniformes **						
** Argentinidae **						
*Argentina aliceae* Cohen & Atsaides, 1969	ICN-MHN 4544; CIRUV 78001; 75002					
** Stomiiformes **						
** Gonostomatidae **						
*Cyclothone microdon* (Günther, 1878)	SIO 55-244, 55-246					Castellanos-Galindo et al. 2006c
*Cyclothone pallida* Brauer, 1902	SIO 55-244;					Castellanos-Galindo et al. 2006c
** Phosichthyidae **						
*Woodsia nonsuchae* (Beebe, 1932)	SIO 55-244					Castellanos-Galindo et al. 2006c
*Yarrella argenteola* (Garman, 1899)	CAS 47830; MCZ 28518; SIO 98-128					Castellanos-Galindo et al. 2006c
** Sternoptychidae **						
*Argyropelecus affinis* Garman, 1899	SIO 55-244, 52-384, 55- 246					Castellanos-Galindo et al. 2006c
** Stomiidae **						
*Astronesthes lampara* Parin & Borodulina, 1998	SIO 03-173, 72-398					Castellanos-Galindo et al. 2006c
*Bathophilus filifer* (Garman, 1899)	ICN-MHN 4559					
*Bathophilus flemingi* Aron & McCrery, 1958	SIO 63-299					Castellanos-Galindo et al. 2006c
*Chauliodus barbatus* Garman, 1899	MCZ 28490; SIO 55-244, 55-246					Castellanos-Galindo et al. 2006c
*Idiacanthus atlanticus* Brauer, 1906	SIO 75-452					Castellanos-Galindo et al. 2006c
*Stomias colubrinus* Garman, 1899	ICN-MHN 4562 - ICN-MHN 4563; CIRUV 94009					
** Ateleopodiformes **						
** Ateleopodidae **						
*Guentherus altivela* Osório, 1917	ROV underwater photography at deep mountains at Malpelo-Yuruparí		**x**		**x**	
** Aulopiformes **						
** Aulopidae **						
*Aulopus chirichignoae* Béarez, Zavalaga & Miranda, 2024	ICN-MHN 4564; CIRUV 017072	Previously identified as *A. bajacali* (Parin & Kotlyar, 1984)				Béarez et al. 2024
** Chlorophthalmidae **						
*Chlorophthalmus mento* Garman, 1899	ICN-MHN 4566; CIRUV 78057; 021227					
** Ipnopidae **						
*Bathypterois atricolor* Alcock, 1896	MCZ 28505, USNM 00057888					Castellanos-Galindo et al. 2006c
*Ipnops agassizii* Garman, 1899	SIO 75-452					Castellanos-Galindo et al. 2006c
** Notosudidae **						
*Scopelosaurus hubbsi* Bertelsen, Krefft & Marshall, 1976	SIO 68-603, 68-616					Castellanos-Galindo et al. 2006c
** Paralepididae **						
*Lestidium bigelowi* Graae, 1967	SIO 96-132, 96-104					Castellanos-Galindo et al. 2006c
** Synodontidae **						
*Synodus evermanni* Jordan & Bollman, 1890	ICN-MHN 4570 - ICN-MHN 4571; CIRUV 950012; 0010042; 006008; 017060; 021245					
*Synodus lacertinus* Gilbert, 1890	CIRUV 79120; 80383; 021267; 023176; 023292					
*Synodus scituliceps* Jordan & Gilbert, 1882	ICN-MHN 4573 - ICN-MHN 4574 - ICN-MHN 4575; CIRUV 80386; 0010043; 0010015; 0010065; 75045; 81226; 81227; 88127; 005116; 81230; 80385; 79121; 81225; 80384; 81228; 81229					
*Synodus sechurae* Hildebrand, 1946	CIRUV 85038					
** Myctophiformes **						
** Myctophidae **						
*Diaphus fulgens* (Brauer, 1904)	SIO 55-244					Castellanos-Galindo et al. 2006c
*Gonichthys cocco* (Cocco, 1829)	LACM 3511, 3515, 50195.001					Castellanos-Galindo et al. 2006c
*Hygophum atratum* (Garman, 1899)	SIO 77-232, 91-189					Castellanos-Galindo et al. 2006c
*Lampanyctus crypticus* (Zahuranec, 2000)	SIO 75-452					Castellanos-Galindo et al. 2006c
*Lampanyctus idostigma* Parr, 1931	SIO 55-244, 52-384, 55- 246					Castellanos-Galindo et al. 2006c
*Lampanyctus omostigma* Gilbert, 1908	SIO 55-242, 55-244, 52- 384					Castellanos-Galindo et al. 2006c
*Notolychnus valdiviae* (Brauer, 1904)	SIO 03-174 55-242, 52- 384					Castellanos-Galindo et al. 2006c
*Parvilux boschmai* Hubbs & Wisner, 1964	SIO 52-384					Castellanos-Galindo et al. 2006c
*Symbolophorus reversus* Gago & Ricord, 2005	SIO 55-242					Castellanos-Galindo et al. 2006c
*Triphoturus mexicanus* (Gilbert, 1890)	ICNMHN 4602, 4603; MCZ 35162; SIO 55-244, 52-384, 55- 246, 55-244;					Castellanos-Galindo et al. 2006c
*Triphoturus nigrescens* (Brauer, 1904)	SIO 55-242, 55-246					Castellanos-Galindo et al. 2006c
** Neoscopelidae **						
*Scopelengys tristis* Alcock, 1890	SIO 55-244, 55-246 CAS 146482					Castellanos-Galindo et al. 2006c
** Lampriformes **						
** Trachipteridae **						
*Zu cristatus* (Bonelli, 1820)	ICN-MHN 4607; Photo Chocó					
**Zeiogadaria**						
** Gadiformes **						
** Bregmacerotidae **						
*Bregmaceros bathymaster* Jordan & Bollman, 1890	ICN-MHN 4623 - ICN-MHN 4624 - ICN-MHN 4625; CIRUV					
** Macrouridae **						
*Asthenomacrurus fragilis* (Garman, 1899)	MCZ 28585					Castellanos-Galindo et al. 2006b
*Coelorinchus canus* (Garman, 1899)	CIRUV 021200					
*Coryphaenoides anguliceps* (Garman, 1899)	MCZ 28565					Castellanos-Galindo et al. 2006b
*Coryphaenoides boops* (Garman, 1899)	MCZ 28588					Castellanos-Galindo et al. 2006b
*Coryphaenoides capito* (Garman, 1899)	MCZ 28591, 28589, 28587					Castellanos-Galindo et al. 2006b
*Coryphaenoides carminifer* (Garman, 1899)	MCZ 28582; USNM 57860					Castellanos-Galindo et al. 2006b
*Nezumia convergens* (Garman, 1899)	MCZ 28574					Castellanos-Galindo et al. 2006b
*Nezumia orbitalis* (Garman, 1899)	MCZ 28578					Castellanos-Galindo et al. 2006b
*Nezumia stelgidolepis* (Gilbert, 1890)	MCZ 28580					Castellanos-Galindo et al. 2006b
** Merlucciidae **						
*Merluccius productus* (Ayres, 1855)	ICN-MHN 4626; CIRUV 021203	Based on morphological and molecular approaches, Silva-Segundo et al. (2011) consider *Merluccius angustimanus* Garman, 1899, a synonym of *M. productus*				
** Moridae **						
*Antimora rostrata* (Günther, 1878)	ROV underwater photography at deep mountains at Malpelo-Yuruparí; MCZ 28610, 28611		**x**		**x**	Castellanos-Galindo et al. 2006b
*Laemonema gracillipes* Garman, 1899	ROV underwater photography at deep mountains at Malpelo-Yuruparí; SU 25629, USNM 00135362		**x**			Castellanos-Galindo et al. 2006b
*Physiculus nematopus* Gilbert, 1890	ICN-MHN 3319					Castellanos-Galindo et al. 2006b
*Physiculus rastrelliger* Gilbert, 1890	CIRUV 80222, 80223					
*Physiculus talarae* Hildebrand & Barton, 1949	ROV underwater photography at deep mountains at Malpelo-Yuruparí; GCRL 13898		**x**		**x**	Castellanos-Galindo et al. 2006b
** Trachichthyiformes **						
** Trachichthyidae **						
*Hoplostethus mento* (Garman, 1899)	INV PEC8840, INV PEC8841					
** Holocentriformes **						
** Holocentridae **						
*Myripristis berndti* Jordan & Evermann, 1903	CIRUV 021326; 021327					
*Myripristis leiognathus* Valenciennes, 1846	CIRUV 79056; 80196; 80197; 91034					
*Neoniphon suborbitalis* (Gill, 1863)	ICN-MHN 4650; CIRUV 010036; 010037; 010038; 011055; 018076					
** Ophidiiformes **						
** Bythitidae **						
*Grammonus diagrammus* (Heller & Snodgrass, 1903)	CIRUV 018225		**x**		**x**	
*Ogilbia sedorae* Møller, Schwarzhans & Nielsen, 2005	USNM 263738					Castellanos-Galindo et al. 2006b
*Petrotyx hopkinsi* Heller & Snodgrass, 1903	ANSP 98557; SIO 70-135					Castellanos-Galindo et al. 2006b
** Ophidiidae **						
*Acanthonus armatus* Günther, 1878	MCZ 28629					Castellanos-Galindo et al. 2006b
*Bassozetus nasus* Garman, 1899	MCZ 28646, 157079					Castellanos-Galindo et al. 2006b
*Bathyonus caudalis* (Garman, 1899)	MCZ 28676, 28678					Castellanos-Galindo et al. 2006b
*Brotula clarkae* Hubbs, 1944	ICN-MHN 4609 - ICN-MHN 4610; CIRUV 0010064; 0010023; 81012; 80058					
*Brotula ordwayi* Hildebrand & Barton, 1949	PE 92008;PE 88078; CIRUV 023070					
*Carapus dubius* (Putnam, 1874)	CIRUV 80090; 81028					
*Carapus mourlani* (Petit, 1934)	CIRUV 023537				**x**	
*Cherublemma emmelas* (Gilbert, 1890)	CIRUV 021221					
*Dicrolene nigra* Garman, 1899	MCZ 28665					Castellanos-Galindo et al. 2006b
*Encheliophis vermicularis* Müller, 1842	USNM 00101790					Castellanos-Galindo et al. 2006b
*Enchelybrotula gomoni* Cohen, 1982	Holotype USNM 221141. Paratypes UF 222859 [ex UMML 22859], USNM 221142, ZMUC P77700	Holotype and paratypes trawled off Chocó, Colombia				
*Holcomycteronus digittatus* Garman, 1899	MCZ 28642, 28640					Castellanos-Galindo et al. 2006b
*Lamprogrammus niger* Alcock, 1891	MCZ 28626- 27; SIO 72-328,					Castellanos-Galindo et al. 2006b
*Lepophidium hubbsi* Robins & Lea, 1978	CIRUV 80256	Material of this cusk-eel stored at CIRUV has been erroneously reported as *L. microlepis* (Gilbert, 1890), a Baja California species. *Lepophidium hubbsi* is known from Costa Rica to Colombia.				
*Lepophidium negropinna* Hildebrand & Barton, 1949	ICN-MHN 4611 - ICN-MHN 4612 - ICN-MHN 4613; CIRUV 78103; 010006; 021046; 021202					
*Lepophidium pardale* (Gilbert, 1890)	IMCN 3340					Castellanos-Galindo et al. 2006b
*Lepophidium prorates* (Jordan & Bollman, 1890)	ICN-MHN 4614; CIRUV 80259; 0010022; 017051; 019019; 021058					
*Neobythites stelliferoides* Gilbert, 1890	IMCN 3342					Castellanos-Galindo et al. 2006b
*Ophidion fulvum* (Hildebrand & Barton, 1949)	ICN-MHN 4616; CIRUV 79075; 79074; 80262; 80263					
*Porogadus longiceps* Garman, 1899	MCZ 28660					Castellanos-Galindo et al. 2006b
**Batrachoidaria**						
** Batrachoidiformes **						
** Batrachoididae **						
*Batrachoides boulengeri* Gilbert & Starks, 1904	CIRUV 3074					
*Batrachoides pacifici* (Günther, 1861)	CIRUV 85073; 88174; 88176; 78008; 80029; 81009; 86029					
*Daector dowi* (Jordan & Gilbert, 1887)	CIRUV 0040100; 0010051; 75003; 001017; 81038; 85003; 85043; 85066; 88051; 88150; 005-112; 88155; 79007; 80032; 70002; 78009; 78010; 79007; 80030; 80031					
*Daector gerringi* (Rendahl, 1941)	CIRUV 89016; 88175			**x**		
*Porichthys margaritatus* (Richardson, 1844)	ICN-MHN 4627; CIRUV 74001; 78011; 78012; 79008; 80033; 81014; 81059; 85094; 85072; 88156; 88052; 88151; 88153; 010026; 021070; 021199					
*Porichthys oculellus* Walker & Rosenblatt, 1988	CIRUV 008-0087					
**Pelagiaria**						
** Scombriformes **						
** Bramidae **						
*Taractes rubescens* (Jordan & Evermann, 1887)	INV PEC 8691					
** Centrolophidae **						
*Schedophilus haedrichi* Chirichigno F., 1973	CIRUV 76004; 87006					
** Gempylidae **						
*Ruvettus pretiosus* Cocco, 1833	Landing picture at Chocó					
** Scombridae **						
*Acanthocybium solandri* (Cuvier, 1832)	Underwater photography at Malpelo Island					Franke and Acero 1992; Acero and Franke 2001
*Auxis brachydorax* Collette & Aadland, 1996	ICN-MHN 4781 - ICN-MHN 4781 – ICN-MHN 4782; CIRUV 90038					
*Euthynnus lineatus* Kishinouye, 1920	ICN-MHN 4783; CIRUV 89058					
*Katsuwonus pelamis* (Linnaeus, 1758)	PE 90122					
*Sarda orientalis* (Temminck & Schlegel, 1844)	PE 88095; 89040					Franke and Acero 1992
*Scomber japonicus* Houttuyn, 1782	INV PEC 124; 634; 881; 884; 6056; 7423.					Franke and Acero 1992
*Scomberomorus sierra* Jordan & Starks, 1895	ICN-MHN 4784 - ICN-MHN 4785; CIRUV 010039; 021086; 021100; 021135; 021233; 021339; 023234					
*Thunnus albacares* (Bonnaterre, 1788)	PE 87081; ICN-MHN 4786					
*Thunnus obesus* (Lowe, 1839)	Landing photographs					
** Stromateidae **						
*Peprilus medius* (Peters, 1869)	CIRUV 78168; 80373; 80374; 81217; 85029; 91008; 021059; 021198					
*Peprilus snyderi* Gilbert & Starks, 1904	CIRUV 71009; 0010058; 021243; 021257					
** Trichiuridae **						
*Trichiurus nitens* Garman, 1899	ICN-MHN 4880 - ICN-MHN 4777 - ICNMHN 4778; CIRUV 7300; 80406; 81235; 88124; 85026; 001008; 010059; 021055; 021101; 021254					
**Syngnatharia**						
** Syngnathiformes **						
** Aulostomidae **						
*Aulostomus chinensis* (Linnaeus, 1766)	Underwater photographs at Gorgona and Malpelo Island					Rubio et al. 1987a; Zapata and Morales 1997; Muñoz and Zapata 2013
** Callionymidae **						
*Synchiropus atrilabiatus* (Garman, 1899)	CIRUV 95020; 017036					
*Synchiropus garthi* (Seale, 1940)	CAS 5746	Holotype collected at Utria, Colombia. Since the holotype is unique and no more specimens have been collected for ~ 100 yrs, we believe this may end up being a synonym of *S. atrilabiatus* but a formal taxonomic study examining the specimen must be performed to reach this conclusion				
** Fistulariidae **						
*Fistularia commersonii* Rüppell, 1838	ICN-MHN 4656; CIRUV 80160; 80161					
*Fistularia corneta* Gilbert & Starks, 1904	ICN-MHN 4657; CIRUV 81091					
** Mullidae **						
*Mulloidichthys dentatus* (Gill, 1862)	ICN-MHN 4731; CIRUV 0010085; 80236; 79069; 80235					
*Pseudupeneus grandisquamis* (Gill, 1863)	CIRUV 71007; 79070; 73001; 80237; 80239; 81123; 81124; 8112; 81126; 81127; 85030; 85050; 011043; 016010; 021082; 021083; 023191					
** Syngnathidae **						
*Cosmocampus coccineus* (Herald, 1940)	ICN-MHN 4650; CIRUV 007-0097					
*Doryrhamphus melanopleura* (Bleeker, 1858)	ICN-MHN 4652 - ICN-MHN 4653; CIRUV Foto Malpelo					
*Hippocampus ingens* Girard, 1858	ICN-MHN 4654; CIRUV 78169; 80375; 80376; 80377; 81220; 81221; 89001; 86028					
*Pseudophallus elcapitanensis* (Meek & Hildebrand, 1914)		Larvae of this freshwater pipefish were reported from material collected off the Colombian Pacific				Beltrán-León et al. 2003
*Pseudophallus starksii* (Jordan & Culver, 1895)		Larvae of this freshwater pipefish were reported from material collected off the Colombian Pacific				Beltrán-León et al. 2003
*Syngnathus auliscus* (Swain, 1882)	ICN-MHN 4655; CIRUV 80379					
**Gobiaria**						
** Kurtiformes **						
** Apogonidae **						
*Apogon atradorsatus* Heller & Snodgrass, 1903	Underwater photography at Malpelo Island		**x**			Muñoz and Zapata 2013
*Apogon dovii* Günther, 1862	ICN-MHN 4674 - ICN-MHN 4675; CIRUV 80008; 80007; 79002					
*Apogon pacificus* (Herre, 1935)	CIRUV 021343					
** Gobiiformes **						
** Eleotridae **						
*Dormitator latifrons* (Richardson, 1844)	ICN-MHN 4752; CIRUV 80506; 79118; 88204.1					
*Eleotris picta* Kner, 1863	CIRUV 80042; 80049; 80056; 81035; 83002; 87002; 88002; 89002; 87017; 90049; 95019; 90035; 91007; 91032; 95050; 86002; 90056; 87017; 91058; 90061; 95021; 88004; 94030; 84002; 94031; 94023; 88004; 83020; 96020; 91069; 93073; 91038; 80380; 79119; 80205; 91032; 94001; 81222; 81223					
*Erotelis armiger* (Jordan & Richardson, 1895)	ICN-MHN 4753; CIRUV 80381; 80382					
*Gobiomorus maculatus* (Günther, 1859)	CIRUV 010135; 010170; 018196; 021133; 012154; 023196; 023213; 023333; 023424					
*Gobiomorus polylepis* Ginsburg, 1953	CIRUV 018197				**x**	
*Guavina micropus* Ginsburg, 1953	CIRUV 018231; 023260				**x**	
** Gobiidae **						
*Aboma etheostoma* Jordan & Starks, 1895	CIRUV 005-060					
*Bathygobius andrei* (Sauvage, 1880)	CIRUV 80174; 85096; 87017; 88167; 88203; 89038; 90058					
*Bathygobius lineatus* (Jenyns, 1841)	CIRUV 015561; 018226					
*Bathygobius ramosus* Ginsburg, 1947	CIRUV 80178; 88187; 80179; 78072; 80175; 80176; 80177; 81102					
*Bollmannia chlamydes* Jordan, 1890	CIRUV 81246					
*Bollmannia* sp. A	CIRUV 021057; 021228	Undescribed species with a known distribution from Panama to Ecuador (Robertson and Allen 2015). Caught in Colombia by deep shrimp trawlers.				
*Bollmannia stigmatura* Gilbert, 1892	CIRUV 017045					
*Cerdale ionthas* Jordan & Gilbert, 1882	CIRUV 006-0031					
*Cerdale paludicola* Dawson, 1974	ICN-MHN 4760; CIRUV 003-0070					
*Chriolepis cuneata* Bussing, 1990	CIRUV 007-0095					
*Chriolepis lepidota* Findley, 1975	CIRUV 018159; 019001; 019004		**x**	**x**		
*Clarkichthys bilineatus* (Clark, 1936)	ICN-MHN 4761 - ICN-MHN 4762; CIRUV 006-003					
*Coryphopterus urospilus* Ginsburg, 1938	PE 89044; PE 91132; CIRUV 023144; 023142; 023336					
*Elacatinus rubrifrons* (Fowler, 1944)	CIRUV 023339; 023348; 023352					
*Evermannia zosterura* (Jordan & Gilbert, 1882)	CIRUV 024065					
*Gobiosoma nudum* (Meek & Hildebrand, 1928)	CIRUV 022015					
*Gobulus crescentalis* (Gilbert, 1892)	CIRUV 007-0096					
*Gobulus hancocki* Ginsburg, 1938	CIRUV 81105; 80185					
*Lophogobius cristulatus* Ginsburg, 1939	CIRUV 018217				**x**	
*Lythrypnus cobalus* Bussing, 1990	CIRUV 018184; 018185; 018186; 018188	Previous records from *L. dalli* (Gilbert, 1890) from Malpelo Island are indeed *L. cobalus*. Molecular and morphological characters support this result.	**x**		**x**	Rojas-Velez 2021
*Lythrypnus solanensis* Acero P., 1981	Holotype LACM 38222-1	A species described based on Colombian material from Chocó		**x**		
*Microdesmus dipus* Günther, 1864	CIRUV 003-001					
*Microdesmus knappi* Dawson, 1972	Holotype USNM 206506. Paratypes ANSP 117497	Holotype and paratypes collected at Punta Guida, Buenaventura, Valle del Cauca		**x**		
*Microdesmus retropinnis* Jordan & Gilbert, 1882	CIRUV 009-0024					
*Microgobius emblematicus* (Jordan & Gilbert, 1882)	CIRUV 71005; 88112; 009-0037					
*Microgobius tabogensis* Meek & Hildebrand, 1928	CIRUV 78074; 80186					
*Ptereleotris carinata* Bussing, 2001	PE 2188; Underwater photographs at Gorgona Island					
*Tigrigobius inornatus* (Bussing, 1990)	CIRUV 023344					
*Tigrigobius janssi* (Bussing, 1981)	CIRUV 023354				**x**	
*Tigrigobius nesiotes* (Bussing, 1990)	Underwater photography at Gorgona Island					
** Oxudercidae **						
*Awaous transandeanus* (Günther, 1861)	CIRUV 86001					
*Ctenogobius sagittula* (Günther, 1862)	CIRUV 018031					
*Evorthodus minutus* Meek & Hildebrand, 1928	CIRUV 009-0036					
*Gobionellus daguae* (Eigenmann, 1918)	Holotype FMNH 58479 [ex CM 7481]. Paratypes CAS 46150 [ex IU 13863], FMNH 58480 [ex CM 7482]	Holotype and paratypes collected at the mouth of Rio Dagua, Valle del Cauca				
*Gobionellus liolepis* (Meek & Hildebrand, 1928)	CIRUV 80180; 80181; 80182; 85041; 88221					
*Sicydium hildebrandi* Eigenmann, 1918	CIRUV 003-082; 008-0067; 008-0103; 020028; 021120; 021121; 021122; 021192; 021193; 023158					
*Sicydium salvini* Ogilvie-Grant,1884	CIRUV 018194					
**Carangaria**						
** Istiophoriformes **						
** Istiophoridae **						
*Istiophorus platypterus* (Shaw, 1792)	Listed in published local inventories with no voucher specimen or photograph					Rubio 1988; Franke and Acero 1996; Acero and Franke 2001
*Makaira nigricans* Lacepède, 1802	Listed in published local inventories with no voucher specimen or photograph					Acero and Franke 2001
** Xiphiidae **						
*Xiphias gladius* Linnaeus, 1758	Listed in published local inventories with no voucher specimen or photograph	Record based on Castellanos-Galindo et al. (2006a) who reported the swordfish from Bahía Málaga				Castellanos-Galindo et al. 2006a
** Carangiformes **						
** Carangidae **						
*Alectis ciliaris* (Bloch, 1787)	ICN-MHN 4684 - ICN-MHN 4685; CIRUV 91021; 80062; 78023; 0010072; 78022					
*Caranx caballus* Günther, 1868	CIRUV 016045; 80059; 78018; 80060; 91018					
*Caranx caninus* Günther, 1867	CIRUV 75005; 80083; 80084; 80085; 81019; 81043; 85016; 79015; 80067; 80068; 78029; 80070; 017008					
*Caranx lugubris* Poey, 1860	Underwater Photographs at Malpelo Island and Chocó					
*Caranx melampygus* Cuvier, 1833	PE 88041; CIRUV 023166; 023475					
*Caranx sexfasciatus* Quoy & Gaimard, 1825	ICN-MHN 4686; CIRUV 018068; 023054; 023407					
*Caranx vinctus* Jordan & Gilbert, 1882	CIRUV 021296	This species is no longer a *Carangoides* (Kimura et al. 2022)				
*Chloroscombrus orqueta* Jordan & Gilbert, 1883	ICN-MHN 4687 - ICN-MHN 4688; CIRUV 88125; 83005; 900054; 91016; 0010011; 85009; 81045; 81058; 85071; 88122; 88050; 016047; 79018; 80076; 80077; 80078; 80079					
*Decapterus macarellus* (Cuvier, 1833)	CIRUV 023101					
*Decapterus macrosoma* Bleeker, 1851	CIRUV 90002; 89063; 75014					
*Elagatis bipinnulata* (Quoy & Gaimard, 1825)	PE 88082; CIRUV 023024; 023339; 023352					
*Euprepocaranx dorsalis* (Gill, 1863)	CIRUV 78034; 81044; 89046; 021261; 022088; 023420	Previously as *Carangoides dorsalis*; Kimura et al. (2022) assigned this species to this new genus				
*Gnathanodon speciosus* (Forsskål, 1775)	Underwater photography at Gorgona Island					Franke and Acero 1993; Muñoz and Zapata 2013
*Hemicaranx leucurus* (Günther, 1864)	CIRUV 78027; 74002					
*Hemicaranx zelotes* Gilbert, 1898	CIRUV 81020; 81046; 88067; 71002; 80071					
*Naucrates ductor* (Linnaeus, 1758)	ICN-MHN 4690; CIRUV 78024					
*Oligoplites altus* (Günther, 1868)	CIRUV 91003; 75007; 79017; 78032; 78033; 80074; 80075; 81023; 81024; 81025; 88194; 91020					
*Oligoplites inornatus* Gill, 1863	ICN-MHN 4691 - ICN-MHN 4692; CIRUV 75004; 85025					
*Oligoplites refulgens* Gilbert & Starks, 1904	CIRUV 81018; 81016; 0010046; 85006; 81017; 85053; 88063; 010066; 010147; 011051; 016050					
*Paraselene orstedii* (Lütken, 1880)	CIRUV 010195; 011018; 016049; 018007; 024090	Previously as *Selene orstedii*; Kimura et al. (2022) assigned this species to this new genus				
*Selar crumenophthalmus* (Bloch, 1793)	ICN-MHN 4693 - ICN-MHN 4694; CIRUV 71001; 78020; 78021; 79117; 80061; 88061; 91064; 010115; 021264; 023046; 023053; 023238					
*Selene brevoortii* (Gill, 1863)	CIRUV 76012; 78028; 80065; 80041; 80088; 81015; 86017; 0010014; 0010038; 016049; 010191; 010171; 023244; 023473; 023115					
*Selene peruviana* (Guichenot, 1866)	ICN-MHN 4695 ICN-MHN 4696 - ICN-MHN 4697; CIRUV 88058; 88060; 75016; 0010040; 0010036; 79013; 80063; 81033; 81048; 81049; 85009; 88139; 005-124; 016048; 004-0169; 80063; 0010024					
*Seriola lalandi* Valenciennes, 1833	ICN-MHN 4698 - ICN-MHN 4699; Photography from Malpelo					
*Seriola peruana* Steindachner, 1881	ICN-MHN 4700; Photography from Malpelo					
*Seriola rivoliana* Valenciennes, 1833	CIRUV 78030; 023483					
*Trachinotus kennedyi* Steindachner, 1876	CIRUV 75001.1; 80064; 81050; 81013; 88138; 88004; 017001; 021291; 023477					
*Trachinotus paitensis* Cuvier, 1832	ICN-MHN 4701; CIRUV 81022; 89066; 023228					
*Trachinotus rhodopus* Gill, 1863	CIRUV 79014; 80069; 010130; 010067; 011016; 017074; 021292; 023045; 023112; 023128; 023408					
*Trachinotus stilbe* (Jordan & McGregor, 1898)	CIRUV 017074; underwater photography at Malpelo Island		**x**			
*Uraspis helvola* (Forster, 1801)	Underwater photographs at Malpelo Island and Chocó					
** Centropomidae **						
*Centropomus armatus* Gill, 1863	CIRUV 80091; 88185; 94004					
*Centropomus medius* Günther, 1864	CIRUV 021093; 023190; 023211					
*Centropomus nigrescens* Günther, 1864	CIRUV 80092; 80093; 81052; 81053; 81054; 89026; 90045					
*Centropomus robalito* Jordan & Gilbert, 1882	CIRUV 017005; 78014; 86010; 90053					
*Centropomus unionensis* Bocourt, 1868	CIRUV 016004					
** Coryphaenidae **						
*Coryphaena equiselis* Linnaeus, 1758	ICN-MHN 4682					
*Coryphaena hippurus* Linnaeus, 1758	ICN-MHN 4680 - ICN-MHN 4681 - ICN-MHN 4683; CIRUV 023422					
** Echeneidae **						
*Phtheirichthys lineatus* (Menzies, 1791)	PE 87067					
*Remora albescens* (Temminck & Schlegel, 1850)	PE 88083; CIRUV 023064					
*Remora osteochir* (Cuvier, 1829)	CIRUV 011066					
*Remora remora* (Linnaeus, 1758)	CIRUV 69001; 79036; 92001; 006021; 023078; 023390					
** Nematistiidae **						
*Nematistius pectoralis* Gill, 1862	CIRUV 005-008; 010168					
** Polynemidae **						
*Polydactylus approximans* (Lay & Bennett, 1839)	ICN-MHN 4720 - ICN-MHN 4721; CIRUV 0010012; 0010035; 005-133; 75037; 79076; 80265; 88187; 005-109; 70006; 71008; 80266; 80267; 80268; 80269; 81144; 85024; 85069; 016034					
*Polydactylus opercularis* (Gill, 1863)	ICN-MHN 4722 ICN-MHN 4723 - ICN-MHN 4724; CIRUV 75038; 80270					
** Sphyraenidae **						
*Sphyraena ensis* Jordan & Gilbert, 1882	ICN-MHN 4769 - ICN-MHN 4770; CIRUV 0010033; 016019; 005-125; 89023; 78164; 74005; 85064; 81213; 0010013					
*Sphyraena idiastes* Heller & Snodgrass, 1903	PE 88126; PE 92068					
*Sphyraena qenie* Klunzinger, 1870	CIRUV 023430					
** Pleuronectiformes **						
** Achiridae **						
*Achirus klunzingeri* (Steindachner, 1880)	ICN-MHN 4799; CIRUV 007-0067; 80361; 80362; 88006; 88145					
*Achirus mazatlanus* (Steindachner, 1869)	ICN-MHN 4800; CIRUV 78159; 80365; 80366; 81120; 86015					
*Achirus scutum* (Günther, 1862)	CIRUV 85054					
*Trinectes fimbriatus* (Günther, 1862)	CIRUV 80359; 80369; 007-0061; 011030; 018053; 018053					
*Trinectes fluviatilis* (Meek & Hildebrand, 1928)	CIRUV 80360; 81207; 81208; 88053; 010123					
*Trinectes fonsecensis* (Günther, 1862)	CIRUV 80369; 80370; 88173; 011029; 016015; 021146					
*Trinectes opercularis* (Nichols & Murphy, 1944)	CIRUV 004-0003; 010086; 010122; 018012; 018059					
*Trinectes xanthurus* Walker & Bollinger, 2001	CIRUV 010090; 011033; 018013; 018038; 022009; 023538					
** Bothidae **						
*Bothus leopardinus* (Günther, 1862)	CIRUV 011058					
*Bothus mancus* (Broussonet, 1782)	Underwater photography at Malpelo Island					
*Monolene maculipinna* Garman, 1899	CIRUV 78045; 021262					
** Cyclopsettidae **		Campbell et al. (2019) included these four genera in their new family Cyclopsettidae, which had already been informally recognized by Betancur et al. (2017)				
*Citharichthys gilberti* Jenkins & Evermann, 1889	ICN-MHN 4793 - ICN-MHN 4794; CIRUV 017011; 94027; 80050; 80054; 88188; 89027; 80051; 80052; 80053; 80055; 78017					
*Citharichthys gnathus* Hoshino & Amaoka, 1999	UCR 1903.002	van der Heiden et al. (2009), in their review of the genus, re-identified two specimens off Guascama, Tumaco, Nariño, as *C. gnathus* previously named “*C. fragilis*”				van der Heiden et al. 2009
*Citharichthys platophrys* Gilbert, 1891	ICN-MHN 4795 - ICN-MHN 4795 - ICNMHN 4796; CIRUV 78041; 79021; 80042; 80043; 80081; 80098					
*Cyclopsetta panamensis* (Steindachner, 1876)	CIRUV 005-071					
*Cyclopsetta querna* (Jordan & Bollman, 1890)	CIRUV 80047; 80046; 88037; 80044; 80045; 88049; 005-127; 72002; 78015; 78016; 79011; 80048; 80049; 0010025					
*Etropus crossotus* Jordan & Gilbert, 1882	ICN-MHN 4797; CIRUV 79022; 80082; 88045; 011183; 018056					
*Etropus peruvianus* Hildebrand, 1946	CIRUV 88046					
*Syacium latifrons* (Jordan & Gilbert, 1882)	CIRUV 81031; 017056					
*Syacium longidorsale* Murakami & Amaoka, 1992	Paratype USNM 317827	Paratype collected off Cauca department				
** Cynoglossidae **						
*Symphurus callopterus* Munroe & Mahadeva, 1989	CAS 24960	Additional non-type specimens in the description				Zapata et al. 1999b; Rojas and Zapata 2006
*Symphurus chabanaudi* Mahadeva & Munroe, 1990	CIRUV 010047					
*Symphurus elongatus* (Günther, 1868)	CIRUV 025072					
*Symphurus fasciolaris* Gilbert, 1892	CIRUV 80128; 81075					
*Symphurus gorgonae* Chabanaud, 1948	ICN-MHN 4803; Gorgona Island, (Pacific) Colombia, depth 30 fathoms. Holotype BMNH 1926.7.12.81. Paratypes BMNH 1926.7.12.82-83	Holotype and paratypes collected at Gorgona island				
*Symphurus melanurus* Clark, 1936	ICN-MHN 4804; CIRUV 78053; 81076; 88054; 011012					
*Symphurus prolatinaris* Munroe, Nizinski & Mahadeva, 1991	CIRUV 78052; 80124; 018035					
*Symphurus undecimplerus* Munroe & Nizinski, 1990	Paratype USNM 304450	Paratype collected south of Punta Ají, Buenaventura, Valle del Cauca				
** Paralichthyidae **						
*Ancylopsetta dendritica* Gilbert, 1890	CIRUV 98006; 88002; 78047; 78046; 90009; 78048; 0010027					
*Hippoglossina bollmani* Gilbert, 1890	CIRUV 76002					
*Hippoglossina montemaris* de Buen, 1961	CIRUV 89059					
*Hippoglossina tetrophthalma* (Gilbert, 1890)	CIRUV 76003; 90010; 021945; 023084					
**Ovalentaria**						
**Incertae sedis in Ovalentaria**						
** Opistognathidae **						
*Lonchopisthus sinuscalifornicus* Castro-Aguirre & Villavicencio-Garayzar, 1988	CIRUV 9007; 00001; 90007; 90059; 91015					
*Opistognathus fenmutis* Acero P. & Franke, 1993	CIRUV 023068; 023374	Bussing and Lavenberg (2003) described four species of *Opistognathus* from the Eastern Pacific Ocean. In their work, both *O. punctatus* Peters, 1869 and *O. scops* (Jenkins & Evermann, 1889) were only included in the key as they were present on Gorgona island and had no support from material examined. Other than this citation, these species have not been reported or collected on the island. Therefore, we decided not to include them here.				
*Opistognathus panamaensis* Allen & Robertson, 1991	ICN-MHN 4671; CIRUV 023523					
**Incertae sedis in Ovalentaria**						
** Pomacentridae **						
*Abudefduf concolor* (Gill, 1862)	ICN-MHN 4732; CIRUV 78106; 0030029					
*Abudefduf troschelii* (Gill, 1862)	CIRUV 87015; 79079					
*Azurina atrilobata* (Gill, 1862)	ICN-MHN 4733; CIRUV 80274					
*Chromis alta* Greenfield & Woods, 1980	Underwater photography at Malpelo Island					Bessudo and Lefevre 2017
*Microspathodon bairdii* (Gill, 1862)	CIRUV 002-0032					
*Microspathodon dorsalis* (Gill, 1862)	CIRUV 79080; 023257; 023258					
*Stegastes acapulcoensis* (Fowler, 1944)	CIRUV 79081; 89065; 021320					
*Stegastes arcifrons* (Heller & Snodgrass, 1903)	Underwater photographs at Gorgona and Malpelo Island; PE					
*Stegastes beebei* (Nichols, 1924)	Underwater photographs at Gorgona and Malpelo Island					
*Stegastes flavilatus* (Gill, 1862)	CIRUV 018003; 021325					
** Atheriniformes **						
** Atherinopsidae **						
*Atherinella pachylepis* (Günther, 1864)	CIRUV 87003; 018039					
*Atherinella panamensis* Steindachner, 1875	CIRUV 011035					
*Atherinella serrivomer* Chernoff, 1986	CIRUV 005-008					
*Atherinella starksi* (Meek & Hildebrand, 1923)	CIRUV 80014					
*Melanorhinus cyanellus* (Meek & Hildebrand, 1923)	Underwater photography at Gorgona Island				**x**	
*Membras gilberti* (Jordan & Bollman, 1890)	CIRUV 80017; 89032; 011036; 023355					
** Beloniformes **						
** Belonidae **						
*Ablennes hians* (Valenciennes, 1846)	PE 91092					Franke and Acero 1996
*Platybelone pterura* (Osburn & Nichols, 1916)	PE 88137; 89052					Franke and Acero 1992
*Strongylura exilis* (Girard, 1854)	CIRUV 80096					
*Strongylura fluviatilis* (Regan, 1903)	CIRUV 010109					
*Strongylura scapularis* (Jordan & Gilbert, 1882)	CIRUV 80034; 80035; 80036; 80037; 86035; 88196; 88099; 89029; 90011; 90034; 011188; 018020; 018021; 018048; 021346; 023210; 023253					
*Tylosurus fodiator* Jordan & Gilbert, 1882	ICN-MHN 4637; CIRUV 005107; 023416					
*Tylosurus melanotus* (Bleeker, 1850)	CIRUV 023372; PE					
*Tylosurus pacificus* (Steindachner, 1876)	CIRUV 89014					
** Exocoetidae **						
*Cheilopogon xenopterus* (Gilbert, 1890)	ICN-MHN 4638; CIRUV 005-233					
*Cypselurus callopterus* (Günther, 1866)	CIRUV 88215; 023011; 023012					
*Exocoetus monocirrhus* Richardson, 1846	CIRUV 88215					
*Fodiator rostratus* (Günther, 1866)	CIRUV 80159					
*Parexocoetus brachypterus* (Richardson, 1846)	PE 87083; CIRUV 018227; 023111					
*Prognichthys sealei* Abe, 1955	ICN-MHN 4639; CIRUV 015546					
** Hemiramphidae **						
*Euleptorhamphus viridis* (van Hasselt, 1823)	CIRUV 023019					
*Hemiramphus saltator* Gilbert & Starks, 1904	CIRUV 80410; 78189					
*Hyporhamphus gilli* Meek & Hildebrand, 1923	CIRUV 85090; 81106; 81107; 80188					
*Hyporhamphus naos* Banford & Collette, 2001	CIRUV 30192; 79055; 83148; 89030					
*Hyporhamphus snyderi* Meek & Hildebrand, 1923	CIRUV 02-014; 03-0051					
*Oxyporhamphus micropterus* (Valenciennes, 1847)	CIRUV 005-232					
** Cyprinodontiformes **						
** Poeciliidae **						
*Poeciliopsis turrubarensis* (Meek, 1912)	CIRUV 011069; 023308; 024066					
** Mugiliformes **						
** Mugilidae **						
*Chaenomugil proboscideus* (Günther, 1861)	CIRUV 93005; 93060; 88018; 80224; 81118; 87005					
*Dajaus monticola* (Bancroft, 1834)	CIRUV 94014; 015547; 019021					
*Mugil cephalus* Linnaeus, 1758	ICN-MHN 4635; CIRUV 81119; 81122; 88174; 88197; 79068; 79067; 80225; 80226; 80227; 80228; 80229; 80230; 81120; 81121; 89034					
*Mugil hospes* Jordan & Culver, 1895	CIRUV 04-0099					
*Mugil setosus* Gilbert, 1892	ICN-MHN 4636; CIRUV 76009; 80231; 80232; 80233; 80234; 88189; 90046	This Eastern Pacific mullet has been widely reported previously as *M. curema* Valenciennes, 1836, an Atlantic species				
** Gobiesociformes **						
** Gobiesocidae **						
*Acyrtus arturo* Tavera, Rojas-Vélez & Londoño-Cruz, 2021	CIRUV -019017; 020011		**x**	**x**		
*Arcos decoris* Briggs, 1969	ICN-MHN 4746; CIRUV 019031					
*Arcos rhodospilus* (Günther, 1864)	CIRUV 006004; CIRUV023530; 023562					
*Gobiesox adustus* Jordan & Gilbert, 1882	CIRUV 80169; 80171; 80170; 81098; 81099					
*Gobiesox daedaleus* Briggs, 1951	CIRUV 015539					
*Gobiesox juradoensis* Fowler, 1944	CIRUV 015550; 018192; 018198; 023332			**x**		
*Gobiesox papillifer* Gilbert, 1890	CIRUV 015562				**x**	
*Tomicodon petersii* (Garman, 1875)	CIRUV 006-008					
*Tomicodon prodomus* Briggs, 1969	CIRUV 81100					
*Tomicodon* sp.	CIRUV 017028			**x**		
*Tomicodon zebra* (Jordan & Gilbert, 1882)	CIRUV 021107				**x**	
** Blenniiformes **						
** Blenniidae **						
*Hypsoblennius brevipinnis* (Günther, 1861)	ICN-MHN 4876 - ICN-MHN 4744; CIRUV 89006; 023167; 023353					
*Hypsoblennius caulopus* (Gilbert, 1898)	CIRUV 89006					
*Hypsoblennius maculipinna* (Regan, 1903)	CIRUV 022013; 022113; 022117					
*Ophioblennius steindachneri* Jordan & Evermann, 1898	CIRUV 75011; 78013; 79010; 80038; 80039; 018071; 021313; 023177					
*Parahypsos piersoni* (Gilbert & Starks, 1904)	CIRUV 80097; 011181					
*Plagiotremus azaleus* (Jordan & Bollman, 1890)	ICN-MHN 4745; CIRUV 015549; 021345; 023155;					
** Chaenopsidae **						
*Acanthemblemaria balanorum* Brock, 1940	CIRUV 025073					Hastings and Robertson 1999
*Acanthemblemaria exilispinus* Stephens, 1963	CIRUV 019018; 023329; 023331					
*Acanthemblemaria hancocki* Myers & Reid, 1936	CIRUV 015560					
*Acanthemblemaria stephensi* Rosenblatt & McCosker, 1988	CIRUV 01544; 018123; 023563			**x**		
*Chaenopsis celeste* Tavera, 2021	CIRUV 019053					
*Chaenopsis deltarrhis* Böhlke, 1957	Holotype CAS-SU 49250 [ex NYZS 28672]. Paratypes ANSP 75217; USNM 101950	Described from Gorgona Island				Böhlke 1957
*Coralliozetus springeri* Stephens & Johnson, 1966	CIRUV 024102					
*Ekemblemaria myersi* Stephens, 1963	CIRUV 0090025; 015553; 023246					
*Emblemaria nivipes* Jordan & Gilbert, 1883	Listed in published local inventories with voucher specimen from Gorgona island, reviewed by Stephens USNM 94020					Stephens 1963
*Protemblemaria bicirrus* (Hildebrand, 1946)	CIRUV 023343; 023345; 024101					
** Labrisomidae **						
*Dialommus macrocephalus* (Günther, 1861)	Photography at Chocó; CIRUV 025074					
*Gobioclinus dendriticus* (Reid, 1935)	CIRUV 91013					
*Malacoctenus ebisui* Springer, 1959	CIRUV 015558; 023330					
*Malacoctenus margaritae* (Fowler, 1944)	CIRUV 018085; 023349					
*Malacoctenus sudensis* Springer, 1959	CIRUV 79026; 80104; 010078; 010082; 020004; 022112					
*Malacoctenus tetranemus* (Cope, 1877)	CIRUV 023350					
*Paraclinus mexicanus* (Gilbert, 1904)	CIRUV 80105; 010081; 018074					
*Starksia fulva* Rosenblatt & Taylor, 1971	CIRUV 008-0109					
*Stathmonotus culebrai* Seale, 1940	CIRUV 024120					
** Tripterygiidae **						
*Axoclinus lucillae* Fowler, 1944	ICN-MHN 4879; CIRUV 0060011					
*Axoclinus rubinoffi* Allen & Robertson, 1992	CIRUV 018147; 18148; 18154		**x**	**x**		
*Lepidonectes bimaculatus* Allen & Robertson, 1992	CIRUV 018134; 018135; 018136; 018137; 018146; 019015		**x**	**x**		
*Lepidonectes clarkhubbsi* Bussing, 1991	Underwater photography at Chocó					
** Eupercaria **						
**Incertae sedis in Eupercaria**						
** Malacanthidae **						
*Caulolatilus affinis* Gill, 1865	ICN-MHN 4677 - ICN-MHN 4678; CIRUV 90017					
*Caulolatilus princeps* (Jenyns, 1840)	ICN-MHN 4679; Photo at Malpelo Island					
*Malacanthus brevirostris* Guichenot, 1848	CIRUV 017079; 023056					
** Pomacanthidae **						
*Holacanthus passer* Valenciennes, 1846	CIRUV 79077; 80271; 021324; 021330					
*Pomacanthus zonipectus* (Gill, 1862)	CIRUV 79078; 85093; 023415; 023417					
** Sciaenidae **						
*Bairdiella ensifera* (Jordan & Gilbert, 1882)	ICN-MHN 4817; CIRUV 017006					
*Corvula macrops* (Steindachner, 1875)	CIRUV 017078					
*Ctenosciaena peruviana* Chirichigno, 1969	CIRUV 021218				**x**	
*Cynoscion albus* (Günther, 1864)	CIRUV 80294; 81163; 81164; 81165					
*Cynoscion analis* (Jenyns, 1842)	CIRUV 73003.1; 88090					
*Cynoscion nortoni* Béarez, 2001	CIRUV 017040; 021208; 023241					
*Cynoscion phoxocephalus* Jordan & Gilbert, 1882	CIRUV 78126; 79088; 80297; 81166; 85036; 79087; 70011; 80296; 80298; 81167; 88091; 88227					
*Cynoscion reticulatus* (Günther, 1864)	ICN-MHN 4818 - ICN-MHN 4819; CIRUV 80299; 81170; 81168; 81169; 83002					
*Cynoscion squamipinnis* (Günther, 1867)	CIRUV 75040; 80300; 80301; 88109; 88111					
*Cynoscion stolzmanni* (Steindachner, 1879)	CIRUV 72007					
*Elattarchus archidium* (Jordan & Gilbert, 1882)	CIRUV 88204; 80303; 88073; 88172					
*Isopisthus altipinnis* (Steindachner, 1866)	ICN-MHN 4725 - ICN-MHN 4726; CIRUV 0010045; 021105; 021147; 021234					
*Larimus acclivis* Jordan & Bristol, 1898	ICN-MHN 4729; CIRUV 016030; 70012; 78127; 88072; 89047; 0010050					
*Larimus argenteus* (Gill, 1863)	CIRUV 81171; 81173; 85059; 88019; 88121; 017003; 75041; 70013; 79091; 80304; 80305; 81172					
*Larimus effulgens* Gilbert, 1898	ICN-MHN 4727 - ICN-MHN 4728; CIRUV 016013; 016023; 016022; 79092; 88035; 88085; 0010097; 0010054; 005130					
*Larimus pacificus* Jordan & Bollman, 1890	CIRUV 78128; 79093					
*Macrodon mordax* (Gilbert & Starks, 1904)	ICN-MHN 4730; CIRUV 78128; 79093					
*Menticirrhus elongatus* (Günther, 1864)	CIRUV 91035; 010120; 021116					
*Menticirrhus nasus* (Günther, 1868)	CIRUV 70014; 81174; 88108; 010070; 010118; 018025					
*Menticirrhus paitensis* Hildebrand, 1946	CIRUV 010073; 018024					
*Menticirrhus panamensis* (Steindachner, 1876)	CIRUV 70015; 79094; 88040; 89020; 004-0143; 75042; 80305; 80306; 80307; 010119; 010111; 023267					
*Micropogonias altipinnis* (Günther, 1864)	CIRUV 79086; 80295					
*Nebris occidentalis* Vaillant, 1897	CIRUV 73003; 78129; 79095; 79096; 81176; 81177; 85058; 81178; 88074; 89018; 01189; 005-018011068; 011047; 012245; 021233					
*Odontoscion xanthops* Gilbert, 1898	CIRUV 78130					
*Paralonchurus dumerilii* (Bocourt, 1869)	CIRUV 76016; 78132; 78133; 80313; 85035; 016035; 018058; 022001					
*Paralonchurus goodei* Gilbert, 1898	CIRUV 010072; 010080; 011023; 016018					
*Paralonchurus petersii* Bocourt, 1869	CIRUV 72010; 78135; 78136; 80315; 80316; 81176; 81183; 88034; 88094; 88081; 010075; 010121; 011024					
*Paralonchurus rathbuni* (Jordan & Bollman, 1890)	CIRUV 88080; 89064					
*Pareques lanfeari* (Barton, 1947)	CIRUV 76017					
*Stellifer chrysoleuca* (Günther, 1867)	CIRUV 72011; 81184; 79101; 80317; 010142					
*Stellifer ephelis* Chirichigno F., 1974	CIRUV 88087; 88093					
*Stellifer ericymba* (Jordan & Gilbert, 1882)	CIRUV 016017; 016020; 79102; 69003; 79103; 80318; 88104; 90060; 006015; 010054; 016017; 016020					
*Stellifer fuerthii* (Steindachner, 1875)	CIRUV 70017; 79104; 80319; 85033; 88021; 88086; 88092; 88135; 88021; 88136; 88137; 88021; 006017; 007106; 010055; 010140; 021117					
*Stellifer illecebrosus* Gilbert, 1898	CIRUV 88017					
*Stellifer imiceps* (Jordan & Gilbert, 1882)	CIRUV 88096; 81179; 011021; 016033	The type species of *Ophioscion*, *O. typicus* Gill, 1863, is phylogenetically nested within the genus *Stellifer* (Chao et al. 2021).				
*Stellifer mancorensis* Chirichigno F., 1962	CIRUV 72012; 79105; 85034; 88106; 010053; 011192; 016019; 021114; 021115; 021187; 021194; 023246; 023297					
*Stellifer melanocheir* Eigenmann, 1918	CIRUV 005-054; 006014; 010052; 011193; 016011; 016012; 023107					
*Stellifer oscitans* (Jordan & Gilbert, 1882)	CIRUV 018027					
*Stellifer pizarroensis* Hildebrand, 1946	CIRUV 85032; 88018					
*Stellifer scierus* (Jordan & Gilbert, 1884)	CIRUV 80310; 81180; 86022; 88016; 89019; 021128; 023055	Previously *Ophioscion. Ophioscion obscurus* Hildebrand, 1946, is a junior synonym of *S. scierus* (Chao et al. 2021).				
*Stellifer strabo* (Gilbert, 1897)	CIRUV 80311; 81181; 89022; 010144; 011022; 021112; 021113					
*Stellifer typicus* (Gill, 1863)	CIRUV 74004; 79098; 80312; 88224; 89021; 004-0111; 010141; 018018; 023042					
*Stellifer zestocarus* Gilbert, 1898	CIRUV 80320; 88105; 81185; 004-064; 006016; 010051; 016016; 023098; 0023243					
*Umbrina analis* Günther, 1868	CAS-ICH 62853	Described as *U. tumacoënsis* Wilson, 1916, a synonym of *U. analis*. Not a type but in a type series				
*Umbrina bussingi* López S., 1980	CIRUV 021260					
*Umbrina dorsalis* Gill, 1862	CIRUV 81187					
*Umbrina xanti* Gill, 1862	CIRUV 70018; 81188; 87022; 017046; 023150					
** Gerreiformes **						
** Gerreidae **						
*Deckertichthys aureolus* (Jordan & Gilbert, 1882)	CIRUV 0010045; 0010020; 88041; 81155					
*Diapterus brevirostris* (Sauvage, 1879)	CIRUV 0010034; 0010021; 80164; 81092; 86011; 88201; 88219; 78066; 78067; 79046; 79047; 80162; 80163; 81093; 88113	*Diapterus peruvianus* (Cuvier, 1830) is considered a synonym of this species. However, Love et al. (2021) and Froese and Pauly (2023) present both species as different.				
*Eucinostomus currani* Zahuranec, 1980	CIRUV 88202; 016032; 018014; 023035; 023236					
*Eucinostomus entomelas* Zahuranec, 1980	CIRUV 016031; 023194					
*Eucinostomus gracilis* (Gill, 1862)	ICN-MHN 4713 - ICN-MHN 4714; CIRUV 78070; 80168; 81097; 88042					
*Eugerres brevimanus* (Günther, 1864)	CIRUV 78071; 85028; 010136; 018045					
*Eugerres lineatus* (Humboldt, 1821)	CIRUV 025075					
*Gerres simillimus* Regan, 1907	ICN-MHN 4715 - ICN-MHN 4716; CIRUV 88159; 88179; 78029					
** Uranoscopiformes **						
** Ammodytidae **						
*Ammodytoides gilli* (Bean, 1895)	CIRUV 86036					
** Uranoscopidae **						
*Kathetostoma averruncus* Jordan & Bollman, 1890	CIRUV 76020; 80801; 80502; 021223					
** Labriformes **						
** Labridae **						
*Bodianus diplotaenia* (Gill, 1862)	ICN-MHN 4736; CIRUV 79058					
*Decodon melasma* Gomon, 1974	CIRUV 91025; 023116					
*Halichoeres aestuaricola* Bussing, 1972	CIRUV 88181; 88182; 011041; 011065; 015543; 023261					
*Halichoeres chierchiae* Di Caporiacco, 1948	Underwater photography at Gorgona Island					Rubio et al. 1987a; Muñoz and Zapata 2013
*Halichoeres discolor* Bussing, 1983	Underwater photography at Malpelo Island		**x**			Bessudo and Lefevre 2017
*Halichoeres dispilus* (Günther, 1864)	CIRUV 75032; 79059; 80203; 80204; 80205; 85027					
*Halichoeres inornatus* (Gilbert, 1890)	Underwater photography at Malpelo Island		**x**			
*Halichoeres malpelo* Allen & Robertson, 1992	CIRUV 018155; 018156; 019016; 019032	Considered endemic, but molecular evidence indicates that it may also be present at Cocos Islands.	**x**			Unpubl. data
*Halichoeres nicholsi* (Jordan & Gilbert, 1882)	CIRUV 022132; 022132					
*Halichoeres notospilus* (Günther, 1864)	CIRUV 79061; 80206; 80207; 80208; 81108					
*Iniistius pavo* (Valenciennes, 1840)	CIRUV 79060					
*Novaculichthys taeniourus* (Lacepède, 1801)	CIRUV 023264					
*Polylepion cruentum* Gomon, 1977	CIRUV 021290					
*Scarus compressus* (Osburn & Nichols, 1916)	Underwater photography at Gorgona Island					
*Scarus ghobban* Fabricius, 1775	CIRUV 78121; 021347; 022122; 023072					
*Scarus perrico* Jordan & Gilbert, 1882	CIRUV 018102; 018112					
*Scarus rubroviolaceus* Bleeker, 1847	CIRUV 018118					
*Stethojulis bandanensis* (Bleeker, 1851)	Underwater photography at Gorgona Island					Zapata and Morales 1997; Muñoz and Zapata 2013
*Thalassoma grammaticum* Gilbert, 1890	Underwater photography at Gorgona Island					Zapata and Morales 1997; Muñoz and Zapata 2013
*Thalassoma lucasanum* (Gill, 1862)	CIRUV 79062; 79063; 80209; 80210; 018079; 019059; 019111; 019113; 021285; 021286; 023103					
** Ephippiformes **						
** Ephippidae **						
*Chaetodipterus zonatus* (Girard, 1858)	ICN-MHN 4765; CIRUV 85045; 78062; 78063; 80152; 80155; 81087; 86013; 0010090; 75027; 90049; 80153					
*Parapsettus panamensis* (Steindachner, 1875)	ICN-MHN 4766; CIRUV 80158; 016036; 88140; 0010088; 81244; 0010096; 79045; 68001; 75028; 78064; 80156; 80157; 81088; 81089; 81090					
** Chaetodontiformes **						
** Chaetodontidae **						
*Chaetodon humeralis* Günther, 1860	CIRUV 86003; 88027; 89002; 01712; 79034; 78054; 78055; 78056; 80131; 81038; 86031					
*Johnrandallia nigrirostris* (Gill, 1862)	CIRUV 75022; 79033; 80129; 80130; 019093; 021283; 023026					
*Prognathodes carlhubbsi* Nalbant, 1995	ROV underwater photography at deep mountains at Malpelo-Yuruparí		**x**			
** Acanthuriformes **						
** Acanthuridae **						
*Acanthurus nigricans* (Linnaeus, 1758)	CIRUV 022121					
*Acanthurus triostegus* (Linnaeus, 1758)	CIRUV 018073; 020005; 022124					
*Acanthurus xanthopterus* Valenciennes, 1835	CIRUV 017010; 022120					
*Ctenochaetus marginatus* (Valenciennes, 1835)	CIRUV 022123; 0030014	Based on molecular evidence this surgeonfish species is nested within the genus *Acanthurus*				Sorenson et al. 2013
*Prionurus laticlavius* (Valenciennes, 1846)	PE 88084; CIRUV 023434					
** Luvaridae **						
*Luvarus imperialis* Rafinesque, 1810	CIRUV 025076					
** Zanclidae **						
*Zanclus cornutus* (Linnaeus, 1758)	CIRUV 79123					
** Lutjaniformes **						
** Haemulidae **						
*Anisotremus caesius* (Jordan & Gilbert, 1882)	CIRUV 021158					
*Anisotremus interruptus* (Gill, 1862)	CIRUV 016003; 81145; 85037; 87001.1					
*Anisotremus taeniatus* Gill, 1861	Underwater photographs at Gorgona Island and Chocó					Tobón-López et al. 2008
*Conodon serrifer* Jordan & Gilbert, 1882	CIRUV 78113; 81149; 88056; 90023					
*Genyatremus dovii* (Günther, 1864)	CIRUV 78107; 78108; 78109; 85055; 016002					
*Genyatremus pacifici* (Günther, 1864)	CIRUV 85046; 81147; 78110; 78111; 78112; 81146					
*Haemulon flaviguttatum* Gill, 1862	Underwater photographs at Gorgona Island and Chocó					
*Haemulon maculicauda* (Gill, 1862)	CIRUV 79083; 89060; 89057					
*Haemulon scudderii* Gill, 1862	CIRUV 016005; 89024					
*Haemulon sexfasciatum* Gill, 1862	CIRUV 80283; 80284; 87010					
*Haemulon steindachneri* (Jordan & Gilbert, 1882)	CIRUV 018092					
*Haemulopsis axillaris* (Steindachner, 1869)	CIRUV 0010055					
*Haemulopsis elongata* (Steindachner, 1879)	CIRUV 023061					
*Haemulopsis leuciscus* (Günther, 1864)	CIRUV 017064; 021129; 022076; 023040					
*Haemulopsis nitida* (Steindachner, 1869)	CIRUV 0010083; 89025; 90041; 80280; 80281; 81148; 81151; 87008; 89044					
*Microlepidotus brevipinnis* (Steindachner, 1869)	CIRUV 017031; 023463					
*Orthopristis chalcea* (Günther, 1864)	CIRUV 78119; 80286; 88163; 016068					
*Pomadasys empherus* Bussing, 1993	CIRUV 017013; 010125; 016074; 18034; 023209; 023250; 023335; 023453	Provisionally retained under *Pomadasys* pending a phylogenetic study: the genus *Pomadasys* has no species in the New World.			**x**	
*Rhencus macracanthus* (Günther, 1864)	CIRUV 81152; 80285; 018066; 018067					
*Rhencus panamensis* (Steindachner, 1876)	CIRUV 0010084; 70009; 78116; 79084; 80122; 80285; 88119; 89010; 005-126; 81154; 78117; 81153; 80285; 88022; 88075; 88168; 90035					
*Rhonciscus bayanus* (Jordan & Evermann, 1898)	CIRUV 94022; 94016; 018105; 018088					
*Rhonciscus branickii* (Steindachner, 1879)	CIRUV 88200; 021060; 023028; 023032; 023445; 023466; 023457					
*Xenichthys xanti* Gill, 1863	CIRUV 70022; 78186; 78187; 78188					
** Lutjanidae **						
*Aphareus furca* (Lacepède, 1801)	Underwater photography at Malpelo Island		**x**			Bessudo and Lefevre 2017
*Hoplopagrus guentherii* Gill, 1862	ICN-MHN 4705; CIRUV 80213					
*Lutjanus aratus* (Günther, 1864)	CIRUV 80214; 81111					
*Lutjanus argentiventris* (Peters, 1869)	CIRUV 95001; 80216; 80225; 89054; 80215; 87014; 91023; 76006					
*Lutjanus colorado* Jordan & Gilbert, 1882	CIRUV 016006; 78084					
*Lutjanus guttatus* (Steindachner, 1869)	ICN-MHN 4706 ICN-MHN 4707 - ICN-MHN 4708; CIRUV 005-0227; 0010081; 001009; 79130; 81112; 85070; 86016; 88186; 85042					
*Lutjanus inermis* (Peters, 1869)	CIRUV 86026; 023033; 023034; 023080; 023255					
*Lutjanus jordani* (Gilbert, 1898)	CIRUV 88015; 010116; 011184; 018004; 018049; 023251					
*Lutjanus novemfasciatus* Gill, 1862	ICN-MHN 4709; CIRUV 016001; 016043					
*Lutjanus peru* (Nichols & Murphy, 1922)	CIRUV 016044; 023428; 023455					
*Lutjanus viridis* (Valenciennes, 1846)	CIRUV 88014; 021328; 021329; 023060					
** Lobotiformes **						
** Lobotidae **						
*Lobotes pacifica* Gilbert, 1898	ICN-MHN 4710 ICN-MHN 4711 - ICN-MHN 4712; CIRUV 81109; 81110; 85091; 015563					
** Spariformes **						
** Sparidae **						
*Calamus brachysomus* (Lockington, 1880)	PE 88096					
** Priacanthiformes **						
** Priacanthidae **						
*Cookeolus japonicus* (Cuvier, 1829)	CIRUV 018249					
*Heteropriacanthus carolinus* (Cuvier, 1829)	CIRUV 023252					
*Pristigenys serrula* (Gilbert, 1891)	ICN-MHN 4672 - ICN-MHN 4673; CIRUV 76012; 023418					
** Lophiiformes **						
** Antennariidae **						
*Abantennarius sanguineus* (Gill, 1863)	ICN-MHN 4631; CIRUV 80005; 79001					
*Antennarius commerson* (Anonymous, 1798)	Underwater photography at Gorgona Island; USNM 207011					Castellanos-Galindo et al. 2006b
*Antennatus strigatus* (Gill, 1863)	CIRUV 0090038					
*Fowlerichthys avalonis* (Jordan & Starks, 1907)	ICN-MHN 4630 CIRUV 91012; 85063; 75001; 76001; 80004; 8000; 91012; 85063; 80003					
** Linophrynidae **						
*Photocorynus spiniceps* Regan, 1925	SIO 69-351, 75-452; ZMUC P 92134;					Castellanos-Galindo et al. 2006b
** Lophiidae **						
*Lophiodes caulinaris* (Garman, 1899)	ICN-MHN 4628 - ICN-MHN 4629; CIRUV 76005; 70007; 71006; 76005; 80211; 80212; 91009; 85082; 76005; 90014; 021209; 021244; 023149					
*Lophiodes spilurus* (Garman, 1899)	CIRUV 95014					
** Ogcocephalidae **						
*Dibranchus erinaceus* (Garman, 1899)	INV PEC5812, INV PEC5814, INV PEC5815, INV PEC8834, INV PEC8835, INV PEC8836, INV PEC8838	The fish identified in INVEMAR collection as *Dibranchus hystrix* Garman, 1899, is actually *D. erinaceus*			**x**	
*Dibranchus nudivomer* (Garman, 1899)	INV PEC8827, INV PEC8828, INV PEC8829, INV PEC8830, INV PEC8831, INV PEC8832				**x**	
*Dibranchus spinosus* (Garman, 1899)	INV PEC8833, INV PEC8837				**x**	
*Ogcocephalus porrectus* Garman, 1899	Underwater photography at Malpelo Island; ROV underwater photography at deep mountains at Malpelo-Yuruparí		**x**			
*Zalieutes elater* (Jordan & Gilbert, 1882)	CIRUV 76010; 78098; 80154; 80248; 80249; 85077; 90008; 021052; 021081; 021110; 021255					
** Oneirodidae **						
*Dolopichthys pullatus* Regan & Trewavas, 1932	NYZC 28769					Castellanos-Galindo et al. 2006b
** Tetraodontiformes **						
** Balistidae **						
*Balistes naufragium* (Jordan & Starks, 1895)	CIRUV 78007; 80027; 80028; 81008; 81611; 85080; 010001; 023425	Santini et al. (2013) in their phylogenetic study demonstrated that this species should not be included in *Pseudobalistes* but in *Balistes*				
*Balistes polylepis* Steindachner, 1876	CIRUV 78006; 81037					
*Canthidermis maculata* (Bloch, 1786)	CIRUV 17024					
*Melichthys niger* (Bloch, 1786)	CIRUV 90040; 0030070; 016077					
*Melichthys vidua* (Richardson, 1845)	Underwater photography at Malpelo Island		**x**			Bessudo and Lefevre 2017
*Sufflamen verres* (Gilbert & Starks, 1904)	ICN-MHN 4809 - ICN-MHN 4810; CIRUV 78025; 80025; 80026; 0030036; 018017; 021097; 022125; 023021; 023133; 023472					
*Xanthichthys caeruleolineatus* Randall, Matsuura & Zama, 1978	Underwater photography at Malpelo Island					Bessudo and Lefevre 2017
*Xanthichthys mento* (Jordan & Gilbert, 1882)	CIRUV 80024; 018016					
** Diodontidae **						
*Chilomycterus reticulatus* (Linnaeus, 1758)	MMG 90154					
*Diodon holocanthus* Linnaeus, 1758	CIRUV 002-003; 016021; 85011					
*Diodon hystrix* Linnaeus, 1758	CIRUV 78043; 023013; 023057					
** Molidae **						
*Mola alexandrini* (Ranzani, 1834)	Landing photograph					
** Monacanthidae **						
*Aluterus monoceros* (Linnaeus, 1758)	CIRUV 81113; 78087; 78088; 79065; 81115; 81014; 81016; 81014					
*Aluterus scriptus* (Osbeck, 1765)	ICN-MHN 4811 - ICN-MHN 4812; CIRUV 79066					
*Cantherhines dumerilii* (Hollard, 1854)	Underwater photography at Malpelo Island					Bessudo and Lefevre 2017
** Ostraciidae **						
*Ostracion meleagris* Shaw, 1796	Underwater photographs at Gorgona and Malpelo Island					Rubio et al. 1987a; Zapata and Morales 1997
** Tetraodontidae **						
*Arothron hispidus* (Linnaeus, 1758)	CIRUV 78170; 80387; 78172; 001005; 80328; 78117; 78170					
*Arothron meleagris* (Anonymous, 1798)	CIRUV 78173					
*Canthigaster janthinoptera* (Bleeker, 1855)	CIRUV 75047					
*Canthigaster punctatissima* (Günther, 1870)	CIRUV 78174; 80392; 003-0015					
*Lagocephalus lagocephalus* (Linnaeus, 1758)	CIRUV 81235; 78176; 81236					
*Sphoeroides annulatus* (Jenyns, 1842)	ICN-MHN 4813; CIRUV 005-101; 79129; 81232; 80393; 80899; 80398; 79125; 78175; 90055; 80400; 80396; 80397; 80394; 86001; 85010; 81233; 75048; 49; 81231; 88025; 005114					
*Sphoeroides lobatus* (Steindachner, 1870)	ICN-MHN 4814 - ICN-MHN 4815 – ICN-MHN 4816; CIRUV 80403; 80402; 0010098; 88026; 84041; 81234; 600989					
*Sphoeroides rosenblatti* Bussing, 1996	CIRUV 90055; 79125; 79124; 005016; 85002; 003110; 005-014; 009070; 89039; 00987; 005045; 005-015; 85012					
*Sphoeroides trichocephalus* (Cope, 1870)	CIRUV 85011; 002-003; 011003; 011067; 011175; 016021; 018047; 021236; 023146					
** Acropomatiformes **						
** Malakichthyidae **						
*Hemilutjanus macrophthalmos* (Tschudi, 1846)	CIRUV 010162	This species has been traditionally identified under Serranidae. Yet, Parenti and Randall (2020) erected a new family (Hemilutjanidae) for it without any formal phylogenetic study. We follow Smith et al. (2022) and place this species in the order Acropomatiformes and the family Malakichthyidae.				
** Centrarchiformes **						
** Cirrhitidae **						
*Cirrhitichthys oxycephalus* (Bleeker, 1855)	CIRUV 79019; 80100; 80101; 80102; 80103; 81248					
*Cirrhitus rivulatus* Valenciennes, 1846	CIRUV 91027					
*Oxycirrhites typus* Bleeker, 1857	Underwater photographs at Gorgona, Malpelo Island and Chocó					
** Kuhliidae **						
*Kuhlia mugil* (Forster, 1801)	CIRUV 80198; 018099; 023123					
** Kyphosidae **						
*Kyphosus elegans* (Peters, 1869)	CIRUV 79057; 021136; 021333; 023121					
*Kyphosus ocyurus* (Jordan & Gilbert, 1882)	CIRUV 80202; 80201; 018100; 023074					
*Kyphosus vaigiensis* (Quoy & Gaimard, 1825)	PE 92075; 023051; 023120					
** Perciformes **						
** Liparidae **						
*Eknomoliparis chirichignoae* Stein, Meléndez C. & Kong U., 1991	CIRUV 95021				**x**	
** Peristediidae **						
*Peristedion barbiger* Garman, 1899	CIRUV 017044; 021210; 023118					
** Scorpaenidae **						
*Pontinus clemensi* Fitch, 1955	CIRUV 80322; 99001; 023437					
*Pontinus furcirhinus* Garman, 1899	CIRUV 010040					
*Pontinus sierra* (Gilbert, 1890)	CIRUV 95003; 010041; 010151; 021231					
*Pontinus* sp. A	ICN-MHN 4658; CIRUV 025077	Undescribed species has a known distribution from Southern Baja California to northern Peru (Robertson and Allen 2015). Frequently caught in Colombia by deep shrimp trawlers.				
*Pontinus strigatus* Heller & Snodgrass, 1903	ROV underwater photography at deep mountains at Malpelo-Yuruparí		**x**		**x**	
*Scorpaena afuerae* Hildebrand, 1946	CIRUV 98005					
*Scorpaena histrio* Jenyns, 1840	CIRUV 80503; 78138; 80325; 81189					
*Scorpaena mystes* Jordan & Starks, 1895	ICN-MHN 4660 - ICN-MHN 4661; CIRUV 78139; 78140; 78159; 80326; 80327; 82001; 023001; 023005; 023122; 023152					
*Scorpaena russula* Jordan & Bollman, 1890	CIRUV 80328; 72013; 80329; 80330; 81190; 86033; 90013					
*Scorpaenodes rubrivinctus* Poss, McCosker & Baldwin, 2010	ROV underwater photography at deep mountains at Malpelo-Yuruparí		**x**		**x**	
*Scorpaenodes xyris* (Jordan & Gilbert, 1882)	ICN-MHN 4659; CIRUV 020001; 020024; 022104; 022105; 022106; 022010; 022014; 022017; 022018; 022019; 023338	A recent article (Victor 2024) presented evidence of two different genetic lineages in the TEP, one from the Galapagos Islands (*S. xyris*) and another from the mainland [*S. chincha* (Nichols & Murphy, 1922)]. We acknowledge this divergence, but in the absence of molecular data and given that we have one population from Malpelo and another from the mainland, we have cautiously decided to retain only *S. xyris* pending further studies.				
** Serranidae **						
*Alphestes immaculatus* Breder, 1936	CIRUV 022020; 022020; 022118					
*Alphestes multiguttatus* (Günther, 1867)	CIRUV 78142; 78143; 90025					
*Anthias noeli* Anderson & Baldwin, 2000	ROV underwater photography at deep mountains at Malpelo-Yuruparí		**x**		**x**	
*Baldwinella eos* (Gilbert, 1890)	CIRUV 021212					
*Cephalopholis colonus* (Valenciennes, 1846)	CIRUV 79113; 80348; 80349; 80350; 80351; 89056					
*Cephalopholis panamensis* (Steindachner, 1876)	ICN-MHN 4663; CIRUV 020026; 021332; 022108					
*Dermatolepis dermatolepis* (Boulenger, 1895)	Underwater photographs at Gorgona and Malpelo Island					Rubio et al. 1987a; Bessudo and Lefevre 2017
*Diplectrum eumelum* Rosenblatt & Johnson, 1974	CIRUV 0010016; 0010032; 0010048; 78145					
*Diplectrum euryplectrum* Jordan & Bollman, 1890	CIRUV 78146					
*Diplectrum labarum* Rosenblatt & Johnson, 1974	CIRUV 016072; 021067					
*Diplectrum macropoma* (Günther, 1864)	CIRUV 77003; 78147; 81193; 81194; 90019; 81195; 80333; 85015; 85067; 88102					
*Diplectrum maximum* Hildebrand, 1946	CIRUV 88103; 023441; 023456					
*Diplectrum pacificum* Meek & Hildebrand, 1925	CIRUV 78148; 70149; 80334; 80335; 88101; 81196; 86021; 90018					
*Diplectrum rostrum* Bortone, 1974	ICN-MHN 4664; CIRUV 78050; 80336; 80337; 81197					
*Epinephelus analogus* Gill, 1863	CIRUV 85014; 78151; 85013; 88077; 0010095; 78152.1; 79108; 80338; 80339; 80340; 81198; 81199; 81200; 81201; 81202; 81203					
*Epinephelus labriformis* (Jenyns, 1840)	CIRUV 79112; 0002; 78153; 78154; 79109; 79110; 79111; 80341; 80342					
*Epinephelus quinquefasciatus* (Bocourt, 1868)	CIRUV 010108; 011037; 018011; 021066					
*Hemanthias peruanus* (Steindachner, 1875)	ICN-MHN 4665; CIRUV 80343; 80344; 80345; 81206; 91029; 80346					
*Hemanthias signifer* (Garman, 1899)	CIRUV 70020; 76018; 80332; 90016					
*Hyporthodus acanthistius* (Gilbert, 1892)	CIRUV 78144; 81191; 88055; 81192; 81247; 86018					
*Hyporthodus cifuentesi* (Lavenberg & Grove, 1993)	CIRUV 023440; 023462; ROV underwater photography at deep mountains at Malpelo-Yuruparí					
*Hyporthodus exsul* (Fowler, 1944)	CIRUV 022011				**x**	
*Hyporthodus mystacinus* (Poey, 1852)	ROV underwater photography at deep mountains at Malpelo-Yuruparí		**x**		**x**	
*Hyporthodus niphobles* (Gilbert & Starks, 1897)	CIRUV 00110047; 80331; 017035; 023019; 023429					
*Liopropoma fasciatum* Bussing, 1980	CIRUV 021298; 023227; 023524					
*Liopropoma longilepis* Garman, 1899	ROV underwater photography at deep mountains at Malpelo-Yuruparí		**x**		**x**	
*Mycteroperca olfax* (Jenyns, 1840)	CIRUV 78156					
*Mycteroperca xenarcha* Jordan, 1888	PE 87045; PE 87025; CIRUV 023254; 023465					
*Paralabrax callaensis* Starks, 1906	CIRUV 023427					
*Paralabrax loro* Walford, 1936	CIRUV 017042					
*Pronotogrammus multifasciatus* Gill, 1863	CIRUV 017054; ROV underwater photography at deep mountains at Malpelo-Yuruparí					
*Pseudogramma thaumasia* (Gilbert, 1900)	CIRUV 80355; 80356; 80357; 83007; 020019; 023159					
*Rypticus bicolor* Valenciennes, 1846	CIRUV 79054; 88062; 88089; 010014; 023263					
*Rypticus nigripinnis* Gill, 1861	ICN-MHN 4666; CIRUV 75030; 78075; 78076; 78077; 78078; 80187; 86014; 85048; 88078; 88207; 89048; 005-157; 010013; 010129; 011006; 011186; 018050; 021225; 023144					
*Serranus psittacinus* Valenciennes, 1846	CIRUV 75044; 78152; 80354; 020023; 021314; 021316; 023522					
*Serranus tico* Allen & Robertson, 1998	CIRUV 023521		**x**			
** Triglidae **						
*Bellator gymnostethus* (Gilbert, 1892)	CIRUV 021230					
*Bellator loxias* (Jordan, 1897)	CIRUV 021050					
*Bellator xenisma* (Jordan & Bollman, 1890)	CIRUV 021051					
*Prionotus albirostris* Jordan & Bollman, 1890	CIRUV 70021					
*Prionotus ruscarius* Gilbert & Starks, 1904	ICN-MHN 4662; CIRUV 78180; 80408; 017033					
*Prionotus stephanophrys* Lockington, 1881	CIRUV 76019; 80403; 81240; 90011; 001001; 021049; 023002; 023009; 023047; 023435; 023436; 023460; 021049					
*Prionotus teaguei* Briggs, 1956	CIRUV 021242					

**Table 2.** Fish species reported north (Panama) and south (Ecuador) of the Colombian marine Pacific waters without vouchered specimens in Colombia.

Lamniformes	Alopiidae	*Alopias vulpinus* (Bonnaterre, 1788)
Lamniformes	Lamnidae	*Isurus oxyrinchus* Rafinesque, 1810
Carcharhiniformes	Sphyrnidae	*Sphyrna zygaena* (Linneaus, 1758)
Torpediniformes	Narcinidae	*Diplobatis ommata* (Jordan y Gilbert, 1890)
Rhinopristiformes	Rhinobatidae	*Pseudobatos planiceps* (Garman, 1880)
Myliobatiformes	Dasyatidae	*Pteroplatytrygon violácea* (Bonaparte, 1832)
Myliobatiformes	Mobulidae	*Mobula mobular* (Bonaterre, 1788)
Myliobatiformes	Mobulidae	*Mobula tarapacana* (Philippi, 1893)
Myliobatiformes	Urotrygonidae	*Urotrygon chilensis* (Günther, 1872)
Myliobatiformes	Urotrygonidae	*Urotrygon reticulata* Miyake & McEachran, 1988
Anguilliformes	Ophichthidae	*Letharchus rosenblatti* McCosker, 1974
Anguilliformes	Ophichthidae	*Ophichthus tetratrema* McCosker & Rosenblatt, 1998
Clupeiformes	Dussumieriidae	*Etrumeus acuminatus* Gilbert, 1890
Clupeiformes	Pristigasteridae	*Opisthopterus macrops* (Günther, 1867)
Scombriformes	Scombridae	*Auxis eudorax Collette & Aadland*, *1996*
Gobiiformes	Gobiidae	*Akko brevis* (Günther, 1864)
Gobiiformes	Gobiidae	*Bollmannia macropoma* Gilbert, 1892
Gobiiformes	Gobiidae	*Bollmannia marginalis* Ginsburg, 1939
Gobiiformes	Gobiidae	*Bollmannia ocellata* Gilbert, 1892
Gobiiformes	Gobiidae	*Gobiosoma paradoxum* (Günther, 186i)
Gobiiformes	Gobiidae	*Gymneleotris seminuda* (Günther, 1864)
Gobiiformes	Gobiidae	*Microgobius crocatus* Birdsong, 1968
Gobiiformes	Gobiidae	*Microgobius curtus* Ginsburg, 1939
Gobiiformes	Gobiidae	*Microgobius miraflorensis* Gilbert & Starks, 1904
Gobiiformes	Oxudercidae	*Gobioides peruanus* (Steindachner, 1880)
Gobiiformes	Oxudercidae	*Gobionellus microdon* (Gilbert, 1892)
Istiophoriformes	Istiophoridae	*Istiompax indica* (Cuvier, 1832)
Istiophoriformes	Istiophoridae	*Kajikia audax* (Philippi, 1887)
Istiophoriformes	Istiophoridae	*Tetrapturus angustirostris* Tanaka, 1915
Carangiformes	Centropomidae	*Centropomus viridis* Lockington, 1877
Carangiformes	Echeneidae	*Remora australis* (Bennett, 1840)
Carangiformes	Echeneidae	*Remora brachyptera* (Lowe, 1839)
Carangiformes	Echeneidae	*Remora osteochir* (Cuvier, 1829)
Pleuronectiformes	Bothidae	*Engyophrys sanctilaurentii* Jordan & Bollman, 1890
Pleuronectiformes	Bothidae	*Perissias taeniopterus* (Gilbert, 1890)
Pleuronectiformes	Cynoglossidae	*Symphurus atramentatus* Jordan & Bollman, 1890
Pleuronectiformes	Cynoglossidae	*Symphurus melasmatotheca* Munroe & Nizinski, 1990
Pleuronectiformes	Cynoglossidae	*Symphurus oligomerus* Mahadeva & Munroe, 1990
Pleuronectiformes	Cyclopsettidae	*Etropus ectenes* Jordan, 1889
Pleuronectiformes	Cyclopsettidae	*Syacium ovale* (Günther, 1864)
Pleuronectiformes	Paralichthyidae	*Paralichthys woolmani* Jordan & Williams, 1897
Beloniformes	Exocoetidae	*Exocoetus volitans* Linnaeus, 1758
Beloniformes	Exocoetidae	*Hirundichthys marginatus* (Nichols & Breder, 1928)
Beloniformes	Exocoetidae	*Hirundichthys rondeletii* (Valenciennes, 1847)
Mugiliformes	Mugilidae	*Mugil thoburni* Jordan & Starks, 1896
Blenniiformes	Blenniidae	*Scartichthys gigas* (Steindachner, 1876)
Blenniiformes	Dactyloscopidae	*Dactyloscopus fallax* Dawson, 1975
Blenniiformes	Dactyloscopidae	*Dactyloscopus fimbriatus* (Reid, 1935)
Blenniiformes	Dactyloscopidae	*Heteristius cinctus* (Osburn & Nichols, 1916)
Blenniiformes	Labrisomidae	*Labrisomus multiporosus* Hubbs, 1953
Incertae sedis in Eupercaria	Sciaenidae	*Cynoscion praedatorius* (Jordan & Gilbert, 1889)
Incertae sedis in Eupercaria	Sciaenidae	*Stellifer vermicularis* (Günther, 1867)
Incertae sedis in Eupercaria	Sciaenidae	*Paralonchurus peruanus* (Steindachner, 1875)
Gerreiformes	Gerreidae	*Eucinostomus dowii* (Gill, 1863)
Uranoscopiformes	Uranoscopidae	*Astroscopus zephyreus* Gilbert & Starks, 1897
Labriformes	Labridae	*Nicholsina denticulata* (Evermann & Radcliffe, 1917)
Tetraodontiformes	Tetraodontidae	*Sphoeroides kendalli* Meek & Hildebrand, 1928
Tetraodontiformes	Tetraodontidae	*Sphoeroides sechurae* Hildebrand, 1946
Perciformes	Triglidae	*Prionotus birostratus* Richardson, 1844

**Table 3.** Fish species reported only from larvae from the Colombian marine Pacific waters.

Order	Familiy	Species
Notacanthiformes	Notacanthidae	*Leptocephalus giganteus* Castle, 1959
Anguilliformes	Congridae	*Paraconger similis* (Wade, 1946)
Anguilliformes	Derichthyidae	*Derichthys serpentinus* Gill, 1884
Anguilliformes	Muraenidae	*Gymnothorax mordax* (Ayres, 1859)
Anguilliformes	Nettastomatidae	*Hoplunnis sicarius* (Garman, 1899)
Anguilliformes	Ophichthidae	*Myrophis vafer* Jordan & Gilbert, 1883
Anguilliformes	Ophichthidae	*Pseudomyrophis micropinna* Wade, 1946
Argentiniformes	Bathylagidae	*Bathylagoides nigrigenys* (Parr, 1931)
Stomiiformes	Gonostomatidae	*Leuroglossus urotranus* Bussing, 1965
Stomiiformes	Gonostomatidae	*Cyclothone acclinidens* Garman, 1899
Stomiiformes	Gonostomatidae	*Cyclothone signata* Garman, 1899
Stomiiformes	Gonostomatidae	*Diplophos proximus* Parr, 1931
Stomiiformes	Phosichthyidae	*Vinciguerria lucetia* (Garman, 1899)
Stomiiformes	Sternoptychidae	*Argyropelecus lychnus* Garman, 1899
Stomiiformes	Sternoptychidae	*Argyropelecus sladeni* Regan, 1908
Stomiiformes	Sternoptychidae	*Maurolicus muelleri* (Gmelin, 1789)
Stomiiformes	Sternoptychidae	*Sternoptyx diaphana* Hermann, 1781
Stomiiformes	Sternoptychidae	*Sternoptyx obscura* Garman, 1899
Stomiiformes	Stomiidae	*Idiacanthus antrostomus* Gilbert, 1890
Aulopiformes	Aulopidae	*Hime* sp.
Aulopiformes	Notosudidae	*Scopelosaurus* sp.
Aulopiformes	Paralepididae	*Lestidiops neles* (Harry, 1953)
Aulopiformes	Paralepididae	*Lestidiops pacificus* (Parr, 1931)
Aulopiformes	Paralepididae	*Stemonosudis macrura* (Ege, 1933)
Aulopiformes	Scopelarchidae	*Rosenblattichthys volucris* (Rofen, 1966)
Aulopiformes	Scopelarchidae	*Scopelarchoides nicholsi* Parr, 1929
Myctophiformes	Myctophidae	*Benthosema panamense* (Tåning, 1932)
Myctophiformes	Myctophidae	*Bolinichthys longipes* (Brauer, 1906)
Myctophiformes	Myctophidae	*Diaphus pacificus* Parr, 1931
Myctophiformes	Myctophidae	*Diogenichthys atlanticus* (Tåning, 1928)
Myctophiformes	Myctophidae	*Diogenichthys laternatus* (Garman, 1899)
Myctophiformes	Myctophidae	*Gonichthys tenuiculus* (Garman, 1899)
Myctophiformes	Myctophidae	*Lampanyctus parvicauda* Parr, 1931
Myctophiformes	Myctophidae	*Loweina rara* (Lütken, 1892)
Myctophiformes	Myctophidae	*Myctophum aurolaternatum* Garman, 1899
Myctophiformes	Myctophidae	*Myctophum nitidulum* Garman, 1899
Myctophiformes	Myctophidae	*Notoscopelus resplendens* (Richardson, 1845)
Myctophiformes	Myctophidae	*Symbolophorus evermanni* (Gilbert, 1905)
Myctophiformes	Myctophidae	*Triphoturus oculeum* (Garman, 1899)
Lampriformes	Trachipteridae	*Trachipterus fukuzakii* Fitch, 1964
Stylephoriformes	Stylephoridae	*Stylephorus chordatus* Shaw, 1791
Gadiformes	Bregmacerotidae	*Bregmaceros* sp.
Gadiformes	Macrouridae	*Trachyrincus helolepis* Gilbert, 1892
Gadiformes	Merlucciidae	*Merluccius productus* (Ayres, 1855)
Beryciformes	Beryciformes	*Melamphaes acanthomus* Ebeling, 1962
Beryciformes	Beryciformes	*Melamphaes* sp. 1
Beryciformes	Beryciformes	*Melamphaes* sp. 2
Beryciformes	Beryciformes	*Scopelogadus bispinosus* (Gilbert, 1915)
Trachichthyiformes	Trachichthyidae	*Hoplostethus pacificus* Garman, 1899
Ophidiiformes	Bythitidae	*Cataetyx simus* Garman, 1899
Ophidiiformes	Ophidiidae	*Carapus mourlani* (Petit, 1934)
Ophidiiformes	Ophidiidae	*Echiodon exsilium* Rosenblatt, 1961
Ophidiiformes	Ophidiidae	*Otophidium indefatigabile* Jordan & Bollman, 1890
Scombriformes	Bramidae	*Brama dussumieri* Cuvier, 1831
Scombriformes	Caristiidae	*Paracaristius nudarcus* Stevenson & Kenaley, 2011
Scombriformes	Chiasmodontidae	*Chiasmodon niger* Johnson, 1864
Scombriformes	Gempylidae	*Gempylus serpens* Cuvier, 1829
Scombriformes	Gempylidae	*Nealotus tripes* Johnson, 1865
Scombriformes	Nomeidae	*Cubiceps pauciradiatus* Günther, 1872
Scombriformes	Nomeidae	*Nomeus gronovii* (Gmelin, 1789)
Scombriformes	Nomeidae	*Psenes sio* Haedrich, 1970
Scombriformes	Trichiuridae	*Lepidopus fitchi* Rosenblatt & Wilson, 1987
Syngnathiformes	Syngnathidae	*Bryx veleronis* Herald, 1940
Kurtiformes	Apogonidae	*Apogon retrosella* (Gill, 1862)
Gobiiformes	Gobiidae	*Gunnellichthys* sp.
Gobiiformes	Gobiidae	*Paragunnellichthys* sp.
Carangiformes	Carangidae	*Decapterus muroadsi* (Temminck & Schlegel, 1844)
Pleuronectiformes	Bothidae	*Bothus constellatus* (Jordan, 1889)
Pleuronectiformes	Cynoglossidae	*Symphurus atramentatus* Jordan & Bollman, 1890
Pleuronectiformes	Cynoglossidae	*Symphurus chabanaudi* Mahadeva & Munroe, 1990
Pleuronectiformes	Cynoglossidae	*Symphurus oligomerus* Mahadeva & Munroe, 1990
Beloniformes	Scomberesocidae	*Cololabis adoceta* Böhlke, 1951
Lophiiformes	Gigantactinidae	*Gigantactis* sp.
Lophiiformes	Lophiiformes	*Borophryne apogon* Regan, 1925
Lophiiformes	Oneirodidae	*Dolopichthys* sp.
Perciformes	Serranidae	*Paralabrax humeralis* (Valenciennes, 1828)
Perciformes	Serranidae	*Serranus aequidens* Gilbert, 1890

Regarding the taxonomical distribution of the Colombian Pacific fish fauna, the 20 most speciose orders are Perciformes (61 spp), Anguilliformes (55), Carangiformes (50), Gobiiformes (47), Pleuronectiformes (38), Lutjaniformes (34), Blenniiformes (32), Ophidiiformes (26), Clupeiformes (25), Myctophiformes (25), Tetraodontiformes (25), Carcharhiniformes (25), Scombriformes (24), Labriformes (22), Stomiatiformes (22), Beloniformes (21), Myliobatiformes (19), Gadiformes (18), Lophiiformes (18), and Aulopiformes (17), accounting for 80% of the total number of species recorded. As for the 20 most species-rich families, the numbers are as follows: Sciaenidae (47), Serranidae (39), Gobiidae (33), Carangidae (32), Myctophidae (24), Haemulidae (23), Labridae (22), Ophidiidae (22), Muraenidae (21), Ophichthidae (16), Ariidae (14), Carcharhinidae (14), Engraulidae (12), Gobiesocidae (12), Lutjanidae (11), Scorpaenidae (11), Chaenopsidae (10), Cyclopsettidae (10), Cynoglossidae (10), and Scombridae (10). Altogether these families account for 47.8% of the total species recorded. On the other hand, 55 families have only one species in the Pacific Ocean of Colombia, seven of those being monotypic, while 21 are represented by only one species in the ETP. The 20 most speciose genera are *Stellifer* (14), *Anchoa* (10), *Gymnothorax* (10), *Symphurus* (10), *Halichoeres* (9), *Lutjanus* (9), *Carcharhinus* (8), *Cynoscion* (7), *Diplectrum* (7), *Caranx* (6), *Centropomus* (6), *Prionotus* (6), *Sphyrna* (6), *Urotrygon* (6), *Cathorops* (5), *Haemulon* (5), *Hyporthodus* (5), *Ophichthus* (5), *Pontinus* (5), *Tomicodon* (5), and *Trinectes* (5). These species together account for 18% of total number of Colombian Pacific species.

The fish families (excluding monotypic ones) that are best represented (as a percentage) in Colombian waters concerning the global and regional (ETP) numbers are as follows. On a global scale, only Coryphaenidae have 100% (2 spp) of their species in the Colombian Pacific. At the regional scale, six families contain 100% of the diversity of ETP species recorded in Colombia. These are Achiridae (8 spp), Sphyrnidae (6 spp), Coryphaenidae (2 spp), and Fistulariidae (2 spp). All these families are lineages in which species diversity can be considered low (>10 spp). After filtering those families with > 10 species and > 20% of the regional richness, 22 families were retained; three have > 80% of the ETP diversity in Colombia and, therefore, can be considered numerous and extremely well represented. Those families are: Carangidae (32 spp: 91.5% of regional diversity), Ophidiidae (22 spp: 87.5% of regional diversity), and Lutjanidae (11 spp: 91.7% of regional diversity). On the other hand, only three families have over 10 species, representing more than 20% of the global diversity of each family (Carangidae 32 spp: 20.4%, Carcharhinidae 14 spp: 23.8%, and Cyclopsettidae 10 spp: 22% of global diversity). Although we recognize that any threshold chosen is arbitrary, we believe that the numbers of these families can be considered high given the inequitable distribution of species in their lineages (i.e., the phylogenetic tree of life is not balanced).

**Figure 2.**
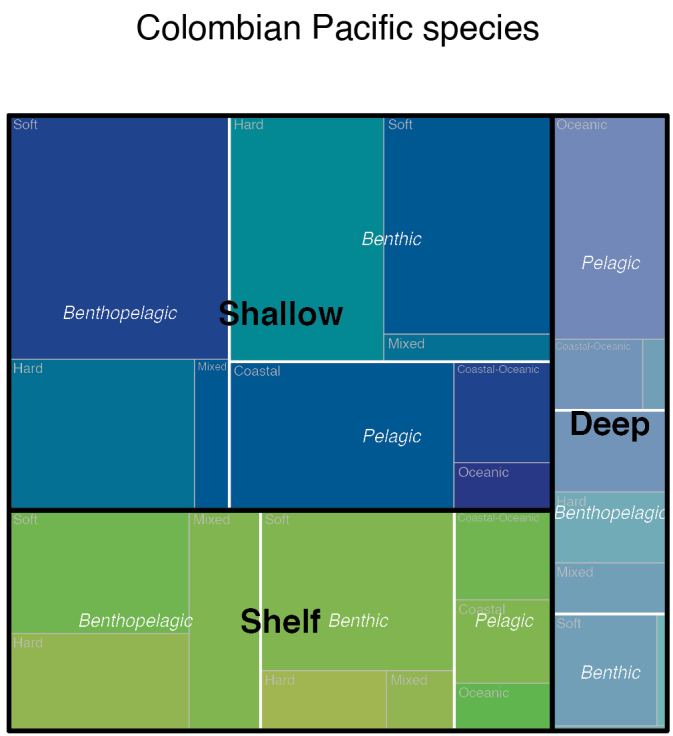
Coarse ecological tree map of Colombian Pacific marine fish fauna. Shallow species correspond to 51%, inhabitants of the continental shelf 28%, and deep waters dwellers 21%.

Analyzing this fauna from a depth and substrate preference perspective, of the 727 species, 32.7% were categorized as benthic, 43.1% as bentho-pelagic, and 24.2% as pelagic according to their vertical position in the water column. Among the pelagic species, 41.8% are coastal, 26.1% coastal-oceanic, and 32% oceanic. Regarding the benthic and benthopelagic species, 54.1% predominantly inhabit soft bottoms, 12.4% are found on soft and hard bottoms, and 33.3% dwell on hard substrates. Regarding depth, 25.9% are associated with deep habitats, 27% are found at intermediate depths, and 46.9% inhabit shallow water. A tree map graph representing the numbers of species into discrete ecological categories according to the environment they inhabit (Fig. 2), allows us to visualize that the dominant ichthyic fauna of the Colombian Pacific is benthic and benthopelagic species related to shallow, soft-bottom environments. Although we recognize that this result is strongly influenced by a sampling bias, specifically in deep waters, an area where work has been very incipient if not almost nonexistent in Colombia, we believe that this trend is truthful given that these environments dominate much of the marine area of the Colombian Pacific, especially the southern region.

In terms of the use of this diversity, 282 species of ray-finned fishes (39% of the total) are considered commercially significant. What is worrying about this data is that most of these species lack species-specific landings data and population studies to assess their conservation status. Of the total number of species, 50 have undergone a national conservation status assessment by the IUCN, which reveals that one specie is Critically Endangered, 13 are Near Threatened, 21 are Vulnerable, two are of Least Concern, and 12 fall into the Data Deficient category (Chasqui et al. 2017). This should serve as a warning to decision makers about the importance of these species and how we must strive to obtain good quality data. This in turn will allow us to know how populations are doing under human pressure and how we can make better decisions to protect them.

## Conclusions

Introduced species, especially from the Caribbean, deserve special attention. This is the case of the silversides *Atherinella chagresi*, *Atherinomorus stipes*, and *Atherina harringtonensis*, which caught our attention as the last two were reported by Robertson and Allen (2024) as being introduced into the Colombian southern Pacific. We looked for evidence of this introduction, and based on our results, it is quite unlikely that these species have established healthy populations in this region. And if, at any time, they had, today there is no evidence of their existence, and we did not include them in the list. An additional introduced species is the cobia *Rachycentron canadum*, which has been reported to occur in the Pacific from Ecuador to Panama (Castellanos-Galindo et al. 2016, 2018; Vega et al. 2016). This species, originally absent from the ETP, was introduced, and reared in the Ecuadorian Pacific at offshore enclosures, with the foreseeable result that many specimens escaped to the wild, and now they are making their way north through Colombia, being found at least to Panama. Whether this species has already been established in the wild is unknown. The case of the tarpon, *Megalops atlanticus*, is quite different. Since 1937 Samuel Hildebrand reported about the presence of the species in the Pacific side of the Panama Canal, which have crossed autonomously from the Caribbean. Neira and Acero (2016) provided information on the presence and abundance of the tarpon in the northern sector (department of Chocó) of the Colombian Pacific. Today, information is available on its wide distribution, reaching as far south as the Mira River, on the border between Colombia and Ecuador. Therefore, the species is well established in the Eastern Pacific, being common at least in Colombian waters.

Overall, this list is a dynamic object that will change as more information becomes available and as some necessary taxonomic revisions occur. We recognize that some species may have been omitted when the samples collected in the Colombian Pacific were deposited in international collections (except for those reviewed in Castellanos-Galindo et al. 2006b, 2006c). However, the objective of this list was to include only records that we could validate. In the future, it will be necessary to revisit all species deposited in museums outside Colombia and include them as future additions to this contribution.

## Additional information

### Use of AI

No use of AI was reported.

### Author ORCIDs

Jose Tavera https://orcid.org/0000-0003-4517-9238

Beatriz S. Beltrán-León https://orcid.org/0000-0002-4399-3642

Andrea Polanco F https://orcid.org/0000-0001-6121-5214

Luis A. Zapata https://orcid.org/0000-0001-5448-888X

Fernando A. Zapata https://orcid.org/0000-0001-8989-4458

Arturo Acero P https://orcid.org/0000-0002-6637-9901

### Data availability

All of the data that support the findings of this study are available in the main text.
